# The *Drosophila* AWP1 ortholog Doctor No regulates JAK/STAT signaling for left–right asymmetry in the gut by promoting receptor endocytosis

**DOI:** 10.1242/dev.201224

**Published:** 2023-03-21

**Authors:** Yi-Ting Lai, Takeshi Sasamura, Junpei Kuroda, Reo Maeda, Mitsutoshi Nakamura, Ryo Hatori, Tomoki Ishibashi, Kiichiro Taniguchi, Masashi Ooike, Tomohiro Taguchi, Naotaka Nakazawa, Shunya Hozumi, Takashi Okumura, Toshiro Aigaki, Mikiko Inaki, Kenji Matsuno

**Affiliations:** ^1^ Department of Biological Sciences, Graduate School of Science, Osaka University, 1-1 Machikaneyama, Toyonaka, Osaka 560-0043, Japan; ^2^ Department of Biological Science and Technology, Tokyo University of Science, 2641 Yamazaki, Noda, Chiba 278-8510, Japan; ^3^ Department of Biological Science, Tokyo Metropolitan University, 1-1 Minami-osawa, Hachioji, Tokyo 192-0397, Japan

**Keywords:** Left–right asymmetry, JAK/STAT signaling, Asymmetric development, Endocytic trafficking, *Drosophila*

## Abstract

Many organs of *Drosophila* show stereotypical left–right (LR) asymmetry; however, the underlying mechanisms remain elusive. Here, we have identified an evolutionarily conserved ubiquitin-binding protein, AWP1/Doctor No (Drn), as a factor required for LR asymmetry in the embryonic anterior gut. We found that *drn* is essential in the circular visceral muscle cells of the midgut for JAK/STAT signaling, which contributes to the first known cue for anterior gut lateralization via LR asymmetric nuclear rearrangement. Embryos homozygous for *drn* and lacking its maternal contribution showed phenotypes similar to those with depleted JAK/STAT signaling, suggesting that Drn is a general component of JAK/STAT signaling. Absence of Drn resulted in specific accumulation of Domeless (Dome), the receptor for ligands in the JAK/STAT signaling pathway, in intracellular compartments, including ubiquitylated cargos. Dome colocalized with Drn in wild-type *Drosophila*. These results suggest that Drn is required for the endocytic trafficking of Dome, which is a crucial step for activation of JAK/STAT signaling and the subsequent degradation of Dome. The roles of AWP1/Drn in activating JAK/STAT signaling and in LR asymmetric development may be conserved in various organisms.

## Teaser

**Summary:** Doctor No, a *Drosophila* AWP1 ortholog, activates JAK/STAT signaling via Dome receptor endocytosis in a crucial step for left–right asymmetry in the developing gut.

## INTRODUCTION

Many animals show directional left–right (LR) asymmetry in their body structure and function. Several mechanisms, such as cilia-generated flow, contribute to LR axis formation in vertebrates (Yoshiba and Hamada, 2014). However, the mechanisms underlying LR asymmetric development in invertebrates are relatively obscure and remain an elemental question in biology (Vandenberg and Levin, 2013).

**Fig. 1.**
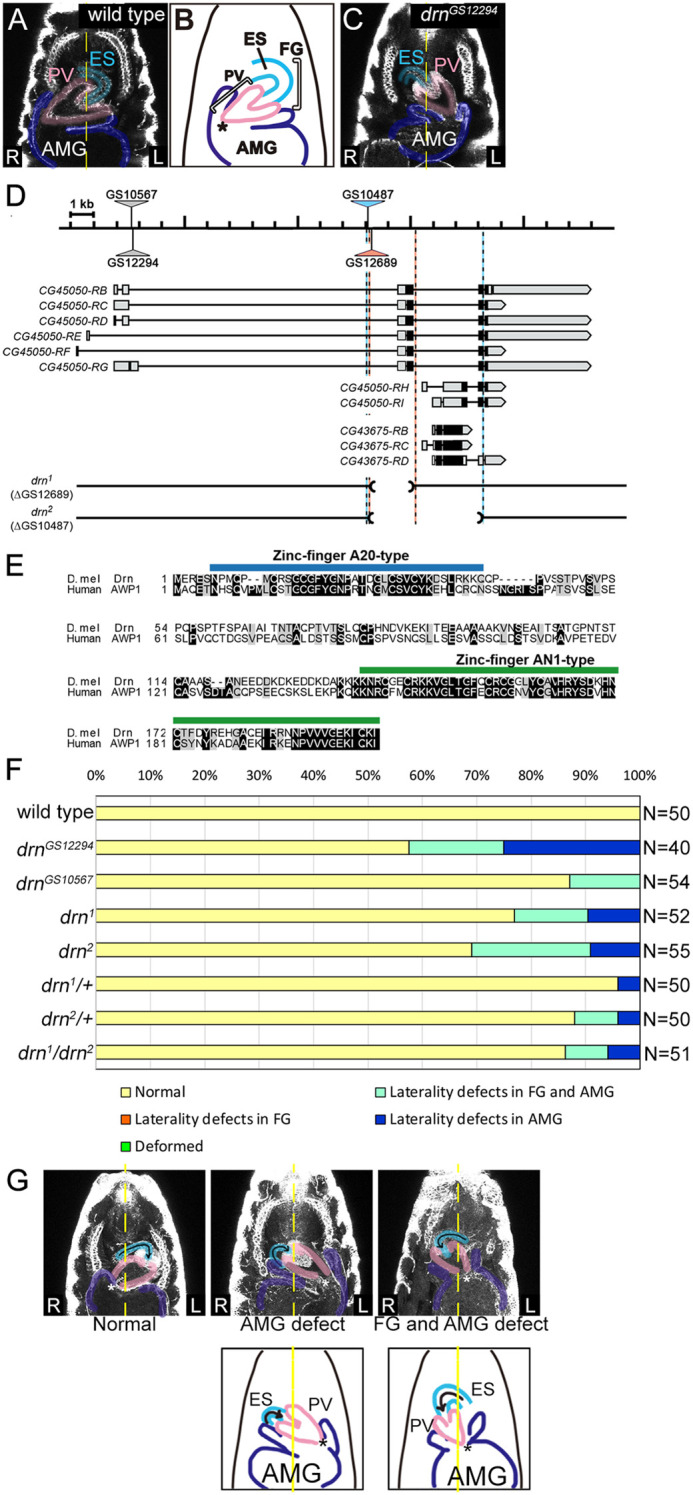
**LR asymmetry of the FG and AMG is defective in *drn* mutants.** (A) Ventral view of a stage 16 wild-type embryo. The gut outline was visualized by anti-Fas3 immunostaining. LR asymmetry of the FG was judged by the direction of ES (turquoise) rotation and that of the AMG was judged by the position of the joint (asterisk in B) between the AMG (dark blue) and PV (pink) relative to the midline (yellow dashed line). (B) Diagram of the FG and AMG in wild-type embryos at stage 16, depicting the ES, PV and AMG. (C) LR inversion of the FG and AMG in *drn^GS12294^* homozygotes. Symbols are the same as those in A. (D) Diagram showing the genomic region of *drn* (top), its alternative transcripts (middle), and the structures of *drn^1^* and *drn^2^* mutants (bottom) with P element insertion sites (triangles), deleted regions (parentheses and dashed lines), exons corresponding to coding regions (black boxes), untranslated regions (gray boxes) and introns (lines). (E) Amino acid sequences of *Drosophila* Drn (NP_001027162.1) and human AWP1 (NP_061879) were optimally aligned using ClustalW. The diagram indicates identical (white characters on black) and similar (black characters on gray) residues. A20-type and AN1-type zinc finger domains are indicated by blue and green lines, respectively. (F) The frequency of FG and AMG LR defects in embryos with the genotypes indicated on the left. Bars denote the percentage of embryos with no laterality defects (yellow), with laterality defects in only the FG (orange) or the AMG (blue), or in both the FG and AMG (turquoise), or with deformities (green). The number (N) of embryos scored is shown on the right. (G) Examples of LR asymmetry phenotypes in the FG and AMG in *drn^2^* homozygotes. Schematics representing LR defects are presented below. The ES (turquoise), PV (pink) and midline (yellow dashed line) are as described in A. L and R represent the left and right sides of embryos, respectively.

Stereotypical LR asymmetry is present in several organs of *Drosophila*, including the gut, testis, male genitalia, and brain (Coutelis et al., 2014; Okumura et al., 2008). Of these organs, the embryonic gut is the first to show LR asymmetry during development (Hayashi and Murakami, 2001; Hozumi et al., 2006). Intriguingly, the anterior and posterior parts of the embryonic gut are controlled by two distinct gene groups. The LR asymmetry of the posterior part is regulated by *Myo1D*, which determines cell chirality (Hozumi et al., 2006; Inaki et al., 2016, 2018; Nakamura et al., 2013; Spéder et al., 2006; Utsunomiya et al., 2019). An entirely different mechanism governs the LR asymmetry of the anterior part. The anterior gut of the embryo consists of the foregut (FG) and midgut (MG), which are complex structures with directional and stereotypical LR asymmetry (Fig. 1A,B) (Hayashi and Murakami, 2001). Various genetic pathways, including the JNK and Wnt pathways, play important roles in LR asymmetric development (Hayashi et al., 2005; Kuroda et al., 2012; Maeda et al., 2007; Okumura et al., 2010; Shin et al., 2021; Taniguchi et al., 2007). A genetic screen performed by our group suggested that the Janus kinase (JAK)/signal transducer and activator of transcription (STAT) signaling pathway is also involved in LR asymmetric development. The present study reveals the involvement of JAK/STAT signaling in LR asymmetric development of the anterior gut.

JAK/STAT signaling, which is evolutionarily conserved from *Drosophila* to humans, is essential for morphogenesis, cell proliferation, cell differentiation, cell death, immunity and other biological events (Arbouzova and Zeidler, 2006; Calò et al., 2003; Lee et al., 2017; Myllymäki and Rämet, 2014; Recasens-Alvarez et al., 2017; Seif et al., 2017). In JAK/STAT signaling in *Drosophila*, the ligands are encoded by *unpaired* (*upd*; *upd1*), *upd2* and *upd3* and the receptor is encoded by *domeless* (*dome*) (Agaisse et al., 2003; Brown et al., 2001; Ghiglione et al., 2002; Harrison et al., 1998; Hombría et al., 2005). JAK is encoded by *hopscotch* (*hop*) in *Drosophila*. Moreover, similar to the mammalian JAK/STAT system, Hop constitutively associates with the intracellular domain of Dome in *Drosophila* (Arbouzova and Zeidler, 2006; Binari and Perrimon, 1994). Ligand binding induces conformational changes in Dome that lead to the phosphorylation of JAK, which phosphorylates Dome to create docking sites for STATs, particularly Stat92E in *Drosophila* (Yan et al., 1996). JAK then tyrosine phosphorylates STATs, which are subsequently dimerized and translocated to the nucleus to bind to enhancers of target genes and activate transcription (Arbouzova and Zeidler, 2006; Hou et al., 1996; Yan et al., 1996) (Fig. 3A).

In mammals, endocytosis regulates JAK/STAT signaling through various mechanisms (German et al., 2011; Lei et al., 2011). Endocytosis and the regulation of JAK/STAT signaling activity are also closely connected in *Drosophila* (Devergne et al., 2007; Moore et al., 2020; Ren et al., 2015; Vidal et al., 2010). In the classic working model of endocytosis, membrane receptors are internalized into endosomal compartments, where they are degraded and recycled. This reduces the number of receptors available to transduce signaling (Cendrowski et al., 2016; Elkin et al., 2016; Piper et al., 2014). Moreover, receptor activity requires endocytic trafficking in some signaling pathways (Cendrowski et al., 2016; Irannejad et al., 2013). Studies on the relationship between endocytosis and JAK/STAT signaling in *Drosophila* have provided contradictory results regarding whether endocytosis upregulates or downregulates JAK/STAT activity (Devergne et al., 2007; Moore et al., 2020; Ren et al., 2015; Vidal et al., 2010). To clarify these conflicting results, it would be helpful to identify and examine a factor that specifically regulates the endocytosis of Dome.

In this study, we found that the *Drosophila* ortholog of AWP1 (associated with PRK1, also known as ZFAND6), which we refer to as Doctor No (Drn), positively regulates JAK/STAT signaling by facilitating endocytic trafficking of the Dome receptor, which is required for normal LR asymmetric development of the embryonic gut. *Drosophila* Drn was named after Ian Fleming's fictional character, whose heart was located on the right side of his chest (Fleming, 1958). Drn protein contains an A20-type zinc finger at the N terminus and an AN1-type zinc finger at the C terminus; this is similar to the mammalian ortholog, which was first identified in humans and mice (Duan et al., 2000). In vertebrates, AWP1 proteins bind to ubiquitin, regulate NF-κB activity, and stimulate the export of Pex5 from the peroxisome, among other roles (Chang et al., 2011; Fenner et al., 2009; Miyata et al., 2012). During *Xenopus* development, AWP1 modifies Wnt and FGF signaling to specify neural crest cells (Seo et al., 2013). However, the molecular mechanisms by which AWP1/Drn proteins influence these various cell signaling pathways are not well understood. In this study, we found that AWP1/Drn plays a crucial role in internalizing the Dome receptor and propose a mechanism by which AWP1/Drn positively modulates JAK/STAT signaling.

## RESULTS

### *drn* mutations affect LR asymmetric gut morphogenesis in *Drosophila*

To identify genes affecting LR asymmetry in the anterior gut, including the FG and anterior MG (AMG), we performed a genetic screen using a large collection of P element insertion lines (*Drosophila* Genes Search Project; http://kyotofly.kit.jp/stocks/documents/GS_lines.html) (Toba et al., 1998). We scored the LR asymmetry phenotypes of the anterior gut in mutants obtained from our genetic screen. The FG is composed of the pharynx, esophagus (ES) and proventriculus (PV), which is a valve-like structure connecting the FG to the AMG (Fig. 1A,B). We defined normal LR asymmetry in the FG and AMG as follows. (1) When viewed from the ventral side, the wild-type *Drosophila* ES loops in an inverse C shape and is connected to the PV (100%, *n*=50); this was defined as normal FG laterality (Fig. 1A). (2) The joint between the PV and AMG is located on the right side of the midline in wild-type embryos (100%, *n*=50); this was defined as normal laterality of the AMG (Fig. 1A). Using these criteria, our genetic screen identified two mutant lines, GS12294 and GS10567, that affect LR asymmetry in the FG and AMG (Fig. 1C).

These lines carry a P element insertion in the *CG45050* locus (Fig. 1D). In this study, we named *CG45050* as *drn*. *Drosophila* Drn and human AWP1 share 42.1% identity and 62.2% similarity in terms of the whole protein (calculated using EMBOSS pairwise alignment algorithms) (Fig. 1E). In particular, an A20-type zinc finger (amino acids 6-40) and an AN1-type zinc finger (amino acids 137-180) are highly conserved (Fig. 1E). A20-type zinc fingers are found in various proteins with ubiquitin-editing functions; these proteins are often associated with human pathogenesis (Heyninck and Beyaert, 1999; Jacque and Ley, 2009; Kim et al., 2021; Rothe et al., 1995; Song et al., 1996). We noted LR asymmetry defects in the anterior gut in 42.5% of *Drosophila* embryos homozygous for *drn^GS12294^* and 13.0% of *Drosophila* embryos homozygous for *drn^GS10567^*, indicating a disturbance in LR asymmetric development (Fig. 1C,F). In contrast, hindgut and posterior MG laterality were normal in all cases examined (*n*=40), indicating heterotaxy but not *situs inversus* in these phenotypes. To genetically characterize *drn* further, we generated deletion mutant alleles of *drn* through imprecise P element excision. In *drn^1^*, the deduced initiation codon and 5′ portion of the coding region were deleted from the *drn* alternative RNA products CG45050-RB, -RC, -RD, -RE, -RF and -RG (Fig. 1D). In *drn^2^*, the deduced initiation codon and most coding sequences were deleted from all *drn* alternative RNA products, suggesting that *drn^2^* is a loss-of-function mutant of *drn* (Fig. 1D). Various degrees of LR asymmetry defects were observed in *drn^1^* or *drn^2^* homozygotes and transheterozygotes, demonstrating that mutations in *drn* are responsible for the LR defect phenotypes (Fig. 1F). These mutant embryos had LR defects in both the FG and AMG or in the AMG alone (orientation of the FG was correct, but the joint between the FG and PV was on the opposite side); however, we did not observe defects in the FG alone (Fig. 1F,G). Thus, LR asymmetry defects in the FG are strictly coupled with those in the AMG, suggesting the primary role of the AMG in LR asymmetric development of the anterior gut, as has been observed in other mutants with defective LR asymmetry (Kuroda et al., 2012; Taniguchi et al., 2007). We also noted relatively mild LR defects in heterozygotes of *drn^1^* or *drn^2^*, indicating that *drn* may behave in a semidominant manner (Fig. 1F).

### *drn* is required for the LR asymmetric rearrangement of circular visceral muscle cells

**Fig. 2.**
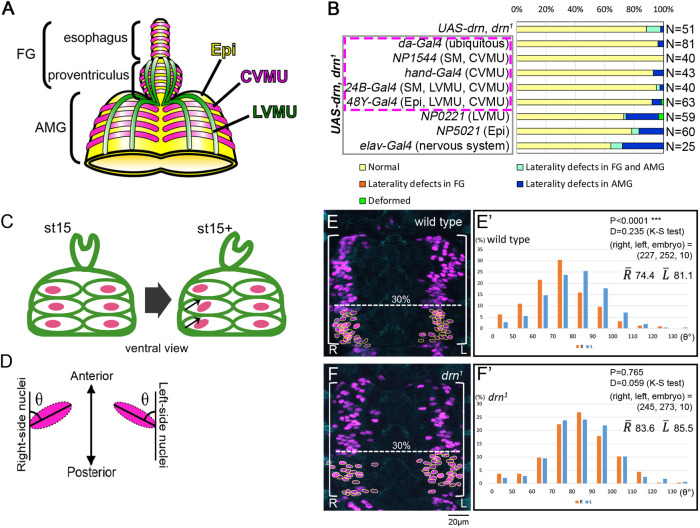
***drn* is required in the CVMU of the midgut for LR asymmetric development of the FG and AMG.** (A) Diagram of the visceral muscles after stage 14: the FG and AMG are overlaid by a layer of CVMU (magenta), and the PV and AMG are covered by LVMU (green). The epithelium (Epi) is shown in yellow. (B) The frequency of LR asymmetry defects in the FG and AMG in embryos carrying *UAS-drn*, *drn^1^*/*drn^1^* without (control, *UAS-drn*, *drn^1^*) or with the GAL4 drivers outlined in gray on the left. *GAL4* expression was driven in the cell types or tissues shown in parentheses (Epi, epithelium of the AMG; SM, somatic muscles). Bars denote the percentage of embryos with no laterality defects (yellow), with laterality defects in only the FG (orange) or the AMG (blue), or in both the FG and AMG (turquoise), or with deformities (green). *UAS-drn* misexpression driven by the GAL4 drivers outlined in magenta rescued the laterality defects. The number (N) of embryos scored is shown on the right. (C) Diagram showing the LR asymmetric rearrangement of nuclei (magenta) in CVMU cells (green ellipses) of the AMG from early (st15) to late (st15+) stage 15. Small arrows indicate the tilt of the ellipsoid nuclei. (D) Diagram showing the major axis angles of the ellipsoid nuclei, represented by the angle (θ) between the major axis of the right- and left-side ellipsoid nuclei and the anterior–posterior axis of the embryo. (E,F) Ventral views of the AMG showing CVMU cells and their nuclei, stained by anti-Fas3 (cyan) and anti-RFP (magenta) antibodies, respectively, in control (E; *65E04-GAL4*/*UAS-RedStinger*) and *drn^1^* homozygous (F; *drn^1^*, *65E04-GAL4*/*drn^1^*, *UAS-RedStinger*) embryos at late stage 15. Nuclei in the lower 30% of the presumptive first chamber (indicated by white brackets) were selected for measurement (encircled by yellow lines). L and R represent the left and right sides of embryos, respectively. Scale bar: 20 μm. (E′,F′) Frequency histograms of the axis angles (in 10° increments) on the left (blue bars) and right (orange bars) sides of CVMU cell nuclei in the ventral AMG of control (E′; *65E04-GAL4*/*UAS-RedStinger*) and *drn^1^* homozygous (F′; *drn^1^*, *65E04-GAL4*/*drn^1^*, *UAS-RedStinger*) embryos at late stage 15. *P*-values (top-right corner) indicate the statistical significance of differences between the angle distributions on the right and left sides calculated using the Kolmogorov–Smirnov test. Numbers in parentheses indicate the numbers (right nuclei, left nuclei and embryos) analyzed. The average angles on the right and left sides are indicated as 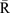 and 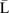, respectively.

To confirm whether *drn* is required for LR asymmetric development of the anterior gut, we performed rescue experiments using the GAL4/UAS system (Brand and Perrimon, 1993). In this system, the expression of wild-type *drn* encoded by *UAS-drn* is driven by various tissue-specific GAL4 drivers in a *drn^1^* homozygote mutant background (Brand and Perrimon, 1993; Elliott and Brand, 2008). The FG and AMG are composed of the epithelium, circular visceral muscle (CVMU) and longitudinal visceral muscle (LVMU) (Fig. 2A). LR defects of the FG and AMG in *drn^1^* homozygotes were reduced by half when *UAS-drn* was introduced without any GAL4 driver (negative control), probably as a result of leaky expression of *UAS-drn* (Figs 1F and 2B). These LR defects were efficiently rescued by *UAS-drn* misexpression driven by *da*-GAL4 (ubiquitous), *NP1522* (in the somatic muscle and CVMU), *hand*-GAL4 (in the CVMU), *24B*-GAL4 (in the CVMU, LVMU and somatic muscle) and *48Y*-GAL4 (in the AMG epithelium, CVMU and LVMU) (Fig. 2B). In contrast, LR defects were not rescued by *UAS-drn* misexpression driven by *NP0221* (in the LVMU), *NP5021* (in the epithelium) or *elav-GAL4* (in the nervous system) (Fig. 2B). Collectively, our data suggest that *drn* expression is primarily required in the CVMU, but not in the LVMU or other tissues for normal LR asymmetric development of the FG and AMG. We also found that in wild-type embryos *UAS-drn* misexpression driven by the GAL4 drivers tested here did not induce marked LR defects in the FG and AMG, suggesting that enhanced *drn* expression did not affect LR asymmetric development in wild-type embryos (Fig. S1).

We previously revealed that the LR asymmetric tilt of the ellipsoidal nuclei in the CVMU cells covering the MG epithelium is the first point of disruption of LR symmetry in the anterior gut (Fig. 2C) (Kuroda et al., 2012; Okumura et al., 2010; Shin et al., 2021; Taniguchi et al., 2007). In wild-type embryos at early stage 15, the major axis of the ellipsoidal nuclei in the CVMU cells was still perpendicular to the midline of the AMG (Fig. 2C). When the MG chambers began dividing at late stage 15, the nuclei in the lower 30% of the presumptive first chamber on the right began tilting diagonally upward and to the right (Fig. 2C). This LR asymmetric nuclear tilt preceded the LR asymmetric morphological changes in the AMG, indicating that it was not a consequence of LR asymmetric morphogenesis (Taniguchi et al., 2007). To analyze whether this process was disrupted in *drn* mutants, we used *65E04-GAL4* to express nuclear-localizing RedStinger fluorescent proteins specifically in the visceral muscles. The angle (θ) between the major axis of the ellipsoidal nuclei in the lower 30% of the presumptive first chamber and the anterior–posterior axis of the embryo was measured by a blind test at late stage 15 (Fig. 2D). Consistent with our previous results, the angle measured was smaller for nuclei on the right side than for those on the left side in wild-type embryos (*P*<0.0001, Kolmogorov–Smirnov test) (Fig. 2E,E′) (Kuroda et al., 2012; Okumura et al., 2010; Taniguchi et al., 2007). However, in *drn^1^* homozygotes, no significant difference was noted in the angle between the left and right sides. Moreover, the angle remained closer to perpendicular on both sides even at late stage 15 (*P*=0.765, Kolmogorov–Smirnov test) (Fig. 2F,F′). In addition to the LR asymmetric tilting of the nuclei, we previously reported that the nuclei assemble in belt-shaped zones along the anterior–posterior axis. These nuclei are scattered in mutants with defects in LR asymmetry of the anterior gut (Shin et al., 2021). In this study, we observed that the distribution of nuclei was more dispersed in *drn^1^* homozygotes than in wild-type embryos in all cases examined (*n*=10) (Fig. 2E-F′). Collectively, these results suggest that *drn* contributes to LR asymmetric development of the AMG by regulating the LR asymmetric rearrangement of nuclei in CVMU cells.

To verify the roles of *drn* in the CVMU, we assessed the distribution of *drn* mRNA in embryos by *in situ* hybridization at various stages of embryogenesis (Fig. S2A-D). Comprehensive analyses of gene expression in *Drosophila* have previously revealed that *drn* is highly expressed in early embryonic stages (Thurmond et al., 2019). In the present study, *drn* mRNA was strongly detected in the preblastoderm to blastoderm (stage 5) stages, suggesting that *drn* mRNA is maternally provided (Fig. S2A,B). We also found that *drn* was broadly expressed at stages 11 and 15, including the trunk visceral mesoderm (TVM, the primordium of the CVMU), in which *drn* is required for LR asymmetric development of the anterior gut (Fig. S2C). In contrast, the negative control (sense probe) exhibited no signal under the same conditions (Fig. S2E-H).

### JAK/STAT signaling is involved in LR asymmetric development of the anterior gut

**Fig. 3.**
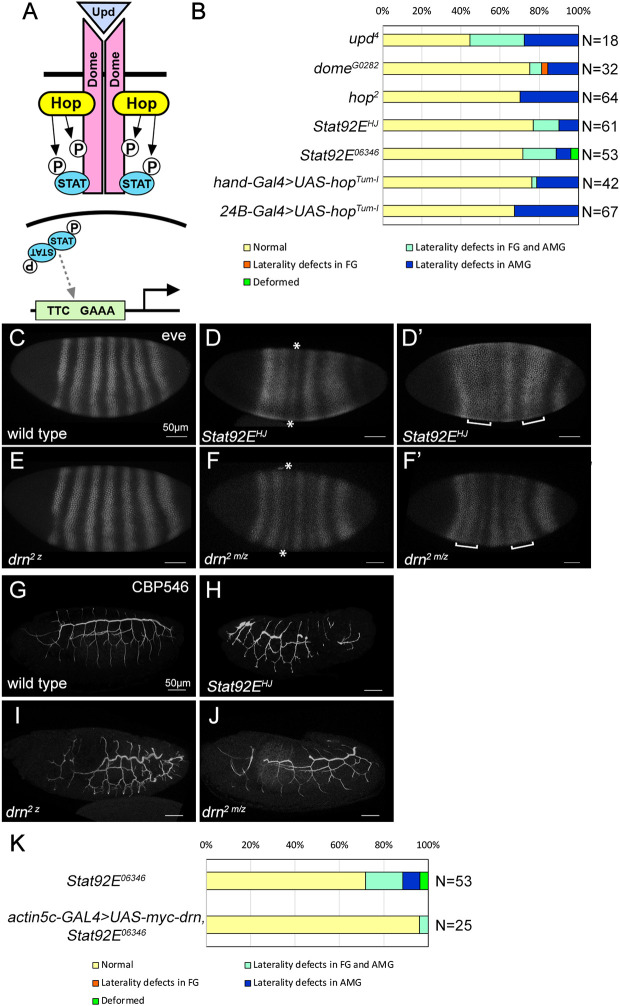
***drn* functions collectively with JAK/STAT signaling during embryonic development.** (A) Illustration of the *Drosophila* JAK/STAT pathway. Dashed gray arrow indicates binding to the enhancer. (B) The frequency of LR asymmetry defects in the FG and AMG of embryos with the genotypes indicated on the left. Bars denote the percentage of embryos with no laterality defects (yellow), with laterality defects in only the FG (orange) or the AMG (blue), or in both the FG and AMG (turquoise), or with deformities (green). The number (N) of embryos scored is shown on the right. (C-F′) The expression of *even-skipped* in wild-type (C), *Stat92E^HJ^* (D,D′), *drn^2 z^* (zygotic mutant) (E) and *drn^2 m/z^* (zygotic and maternal mutant) (F,F′) embryos. D and F show the phenotype of weakened stripe 3 (marked with asterisks). D′ and F′ show the phenotype of stripe fusion (marked with brackets). (G-J) The trachea was visualized by CBP546 staining in wild-type (G), *Stat92E^HJ^* (H), *drn^2 z^* (zygotic mutant) (I) and *drn^2 m/z^* (zygotic and maternal mutant) (J) embryos. (K) The frequency of LR asymmetry defects observed in the FG and AMG of *Stat92E^06346^* homozygotes without (control, top) and with ubiquitous misexpression of *UAS-myc-drn* driven by *actin5c-GAL4* (bottom). Bars show the percentage of embryos with normal laterality (yellow), laterality defects in both the FG and AMG (turquoise), laterality defects in only the AMG (blue), or deformities (green). The number (N) of embryos scored is shown on the right. Scale bars: 50 μm.

Although previous research has suggested that Drn negatively regulates JAK/STAT signaling in cultured *Drosophila* cells (Vidal et al., 2010), the role of Drn has not been explored further *in vivo*. As *drn* is involved in JAK/STAT signaling, we hypothesized that JAK/STAT signaling plays a role in LR asymmetric morphogenesis of the anterior gut. Various mutants of genes involved in the JAK/STAT signaling pathway, including *dome*, *Stat92E*, *upd* and *hop*, were scored for LR phenotypes (Fig. 3B). The roles of these gene products are presented schematically in Fig. 3A. These mutants showed various degrees of LR defects in the FG and AMG, indicating that JAK/STAT signaling is indispensable for normal LR asymmetric development of the anterior gut (Fig. 3B). In addition, when JAK/STAT signaling was augmented by misexpression of an activated form of Hop (*UAS-hop^Tum-l^*) (Harrison et al., 1995), specifically in CVMU cells under the control of *hand-GAL4* or *24B-GAL4*, the embryos showed LR defects in the FG and AMG (Fig. 3B). Therefore, excessive activation of JAK/STAT signaling can also disrupt the LR asymmetry of the FG and AMG. Taken together, these findings indicate that JAK/STAT signaling activity must be maintained at proper levels for LR asymmetric development of the anterior gut.

### *drn* is a general component of the JAK/STAT signaling pathway

To analyze the connection between *drn* and JAK/STAT signaling more directly, we assessed the characteristic phenotypes related to JAK/STAT signaling in *drn* mutants. The expression of a pair-rule gene, *even-skipped* (*eve*), was disturbed in embryos homozygous for *Stat92E*, such as *Stat92E^HJ^*, with approximately 67% of the embryos showing aberrant phenotypes, such as weak stripe 3 (19%), fusion between stripes 2 and 3 (19%), or fusion between stripes 5 and 6 (67%) (*n*=27), rather than the seven-stripe pattern noted in wild-type embryos (Fig. 3C-D′) (Yan et al., 1996). We next analyzed the expression of *eve* in embryos homozygous for *drn^2^* and found that all embryos had normal *eve* expression and displayed a normal seven-stripe pattern (*n*=20) (Fig. 3E). As we found that the mRNA of *drn* is maternally supplied (Fig. S2A,B), we genetically removed maternal *drn* from *drn^2^* homozygotes (*drn^2 m/z^*). The phenotypes of *drn^2 m/z^* embryos were similar to those of *Stat92E^HJ^* homozygotes (36%, *n*=33) (Fig. 3F,F′). These results suggest that *drn* is required for JAK/STAT signaling, with Stat92E playing an essential role. To verify this idea, we examined trachea morphology, which is controlled by JAK/STAT signaling in later embryonic stages (stages 15-17) (Li et al., 2003). The trachea was detected by CBP546 staining (Dong et al., 2014). In *Stat92E^HJ^* homozygotes, the dorsal trunk of the trachea was disturbed compared with that in wild-type embryos (Fig. 3G,H). Moreover, it was truncated in some *drn^2^* homozygotes (35%, *n*=20) and *drn^2 m/z^* embryos (33%, *n*=21). Defects in *drn^2 m/z^* embryos were more severe than those in *drn^2 z^* homozygotes, which can be predicted from the maternal contribution of *drn* (Fig. 3I,J). These results suggested that, similar to *Stat92E*, *drn* plays a positive role in JAK/STAT signaling during embryonic development.

To verify this possibility, we assessed whether the ubiquitous misexpression of *drn*, driven by *actin5c-GAL4*, could rescue LR defects in embryos homozygous for *Stat92E^06346^*. The embryos homozygous for *Stat92E^06346^* showed LR defects in the anterior gut at a frequency of 25% (Fig. 3K); however, the ubiquitous misexpression of *drn* reduced the frequency of LR defects to 4% (Fig. 3K). This result can be explained by a previous finding that *Stat92E* exhibits a maternal effect (Hou et al., 1996; Li et al., 2003; Tsurumi et al., 2011). We speculated that the supply of maternal *Stat92E* to *Stat92E^06346^* mutant embryos is sufficient to support the activity of overexpressed *drn*, which can consequently rescue LR defects of these embryos. In particular, our studies demonstrate that *drn* positively contributes to the activation of JAK/STAT signaling in three different developmental contexts. Therefore, we propose that Drn is a general component positively acting on JAK/STAT signaling, although a previous study involving knockdown by RNA interference (RNAi) and a reporter assay revealed that wild-type *drn* could downregulate JAK/STAT signaling in cultured *Drosophila* cells (Vidal et al., 2010). The cause of this discrepancy is unclear, but may involve a negative feedback loop that is active in a particular time frame during JAK/STAT signaling, as detected by the reporter assay in cultured cells.

### Drn partially localizes to various compartments of endocytic pathway

Although the biochemical roles of Drn have not been studied in *Drosophila*, the mammalian ortholog AWP1 binds to ubiquitin and modulates the functions of ubiquitylated proteins in mammals (Chang et al., 2011; Duan et al., 2000; Fenner et al., 2009). This process is often related to endocytic trafficking and the lysosomal breakdown of membrane receptors (Piper et al., 2014). Thus, we hypothesized that Drn is involved in endocytic trafficking that regulates the JAK/STAT signaling pathway. As TVM and CVMU cells are located deep inside the embryo, it is difficult to obtain clear microscopic images, making them unsuitable for analyzing the subcellular localization of Drn. However, *in situ* hybridization analysis revealed that *drn* is also expressed in the epidermis of the embryo (Fig. S2). Hence, we analyzed the potential colocalization of Drn with various endocytic compartments in the epidermis.

**Fig. 4.**
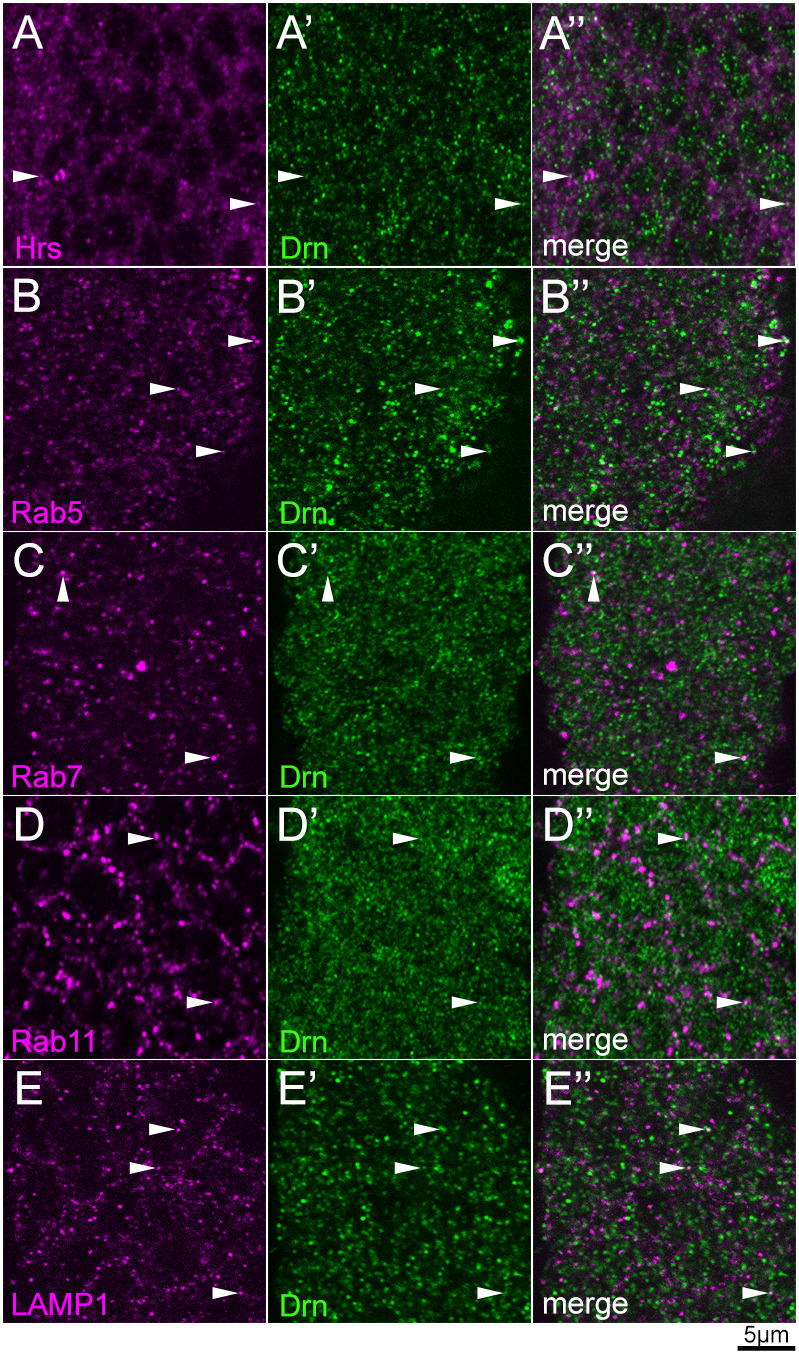
**Drn occasionally colocalizes with markers of various endocytic compartments in the epidermis of wild-type embryos.** (A-E″) Drn subcellular localization in the epidermal cells of wild-type embryos. Embryos were double-stained with anti-Drn (green; middle and right columns) and the following endosomal markers (magenta; left and right columns): (A,A″) Hrs (early endosomes), (B,B″) Rab5 (early endosomes), (C,C″) Rab7 (late endosomes), (D,D″) Rab11 (recycling endosomes) and (E,E″) LAMP1 (lysosomes). A″-E″ show merged images of A-E and A′-E′, respectively. White arrowheads indicate vesicles showing the colocalization of Drn with markers of various endocytic compartments. Scale bar: 5 μm.

To detect Drn protein, we generated a polyclonal antibody (anti-Drn antibody) against full-length Drn and assessed its specificity using the UAS-GAL4 system and RNAi against *drn* in order to deplete Drn in the stripe along the anterior–posterior boundary of the wing disc (the region expressing *ptc*). We found that anti-Drn antibody staining was largely absent from the stripe, confirming the specificity of the antibody (Fig. S3). Using this anti-Drn antibody, we examined the potential colocalization of Drn with various endosomal compartments in the epidermis of wild-type embryos. Drn was detected as punctae in the cytosol and was occasionally found colocalized with endosomal markers, such as Hrs (early endosomes), Rab5 (early endosomes), Rab7 (late endosomes), LAMP1 (lysosomes) and Rab11 (recycling endosomes) (white arrowheads in Fig. 4). Thus, although the distribution of Drn did not concentrate with any particular endosomal markers, Drn appeared to localize with endocytic compartment markers, such as Hrs, Rab5, Rab7 and LAMP1, at a low frequency; this is consistent with our hypothesis that Drn plays some role in endocytic trafficking.

### Drn is required for endocytic trafficking of the Dome receptor

**Fig. 5.**
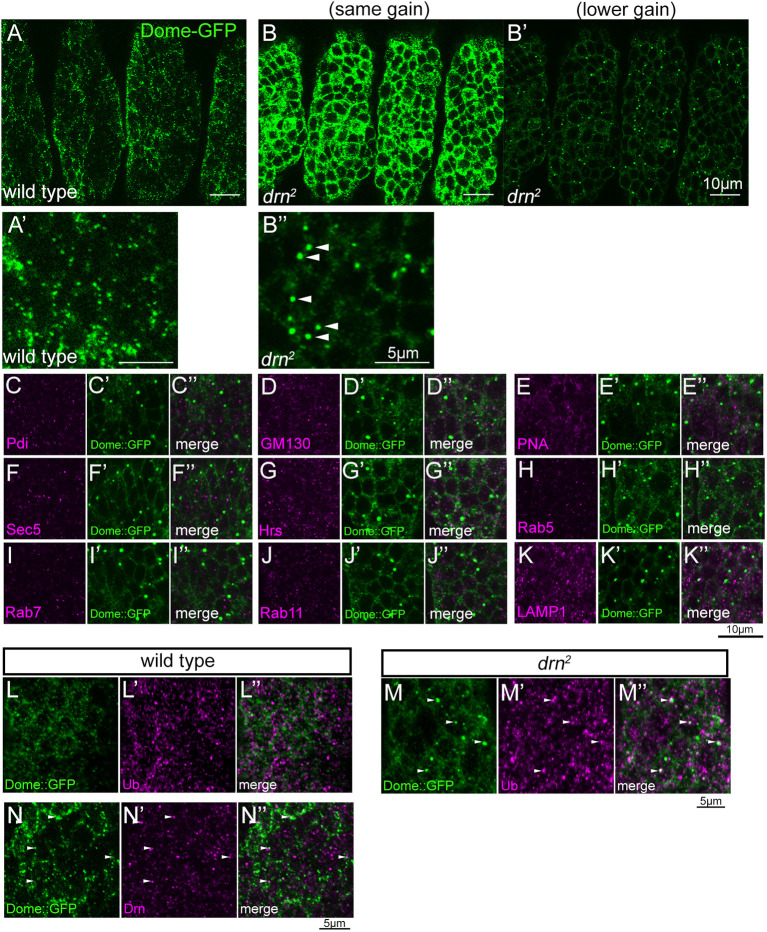
**Dome accumulates in the cell cortex and intracellular structures in the epidermis of *drn* mutant embryos.** (A-B″) Ubiquitous *UAS-dome-GFP* expression was driven by *da-GAL4.* Dome-GFP was detected by anti-GFP antibody staining in the epidermal cells of wild-type (A,A′) and *drn^2^* (B-B″) embryos. Gain to capture was the same for images in A, A′ and B. A lower gain was used for images in B′ and B″ to visualize intracellular aggregations and avoid signal saturation. A′ and B″ are magnified views of A and B′, respectively. White arrowheads in B″ indicate Dome-GFP aggregations. Scale bars: 10 μm (A-B′); 5 μm (A′,B″). (C-K″) The subcellular localization of Dome-GFP aggregates in epidermal cells of *drn^2^* mutants. Embryos were double-stained with anti-GFP antibody (green; middle and right columns) and antibodies against the following markers of intracellular compartments (magenta; left and right columns): (C,C″) Pdi (endoplasmic reticulum), (D,D″) GM130 (*cis*-Golgi), (E,E″) PNA (*trans*-Golgi), (F,F″) Sec5 (exocyst), (G,G″) Hrs (early endosomes), (H,H″) Rab5 (early endosomes), (I,I″) Rab7 (late endosomes), (J,J″) Rab11 (recycling endosomes) and (K,K″) LAMP1 (lysosomes). C″-K″ show merged images of C-K and C′-K′, respectively. Scale bar: 10 μm. (L-M″) The expression of *UAS-dome-GFP* was ubiquitously driven by *da-GAL4* in wild-type (L-L″) and *drn^2^* mutant (M-M″) embryos. Dome-GFP and ubiquitylated proteins were detected by anti-GFP (green; L,L″,M,M″) and anti-ubiquitin (Ub) (magenta; L′,L″,M′,M″) antibody staining, respectively, in epidermal cells. L″ and M″ are merged images of L,L′ and M,M′, respectively. White arrowheads denote vesicles showing colocalization between aggregated Dome-GFP and Ub in *drn^2^*. Scale bar: 5 μm. (N-N″) The expression of *UAS-dome-GFP* was ubiquitously driven by *da-GAL4* in the wild type. Dome-GFP and Drn were detected by anti-GFP (green; N,N″) and anti-Drn (magenta; N′,N″) antibody staining, respectively, in epidermal cells. N″ is a merged image of N and N′. White arrowheads denote vesicles showing colocalization between Dome-GFP and Drn. Scale bar: 5 μm.

As Drn positively contributes to JAK/STAT signaling by regulating endocytic trafficking, we speculated that Drn regulates the JAK/STAT signaling receptor Dome during its endocytic trafficking (Brown et al., 2001). We analyzed the subcellular distribution of Dome using full-length Dome protein that had a C-terminal GFP tag (Dome-GFP) and maintained wild-type Dome functions (Ghiglione et al., 2002). *UAS-dome-GFP* was ubiquitously driven under the control of *da-GAL4* in wild-type and *drn* homozygous embryos (Fig. 5A-B″).

In wild-type embryos, Dome-GFP was detected in epidermal cells as punctae localized to the vicinity of the plasma membrane and cytosolic vesicles (Fig. 5A,A′; Fig. S4). Cytoplasmic vesicles containing Dome-GFP were occasionally labeled using markers of intracellular components, such as Rab5, Rab11 and LAMP1; however, Dome-GFP did not appear to associate with any particular intracellular component (Fig. S4). Previously, Dome was found to accumulate in ubiquitylated cargoes when endocytosis was impaired (Tognon et al., 2014). Hence, we assessed whether these vesicles containing Dome-GFP were stained with an anti-ubiquitin antibody that could recognize mono- and polyubiquitin. However, we did not detect colocalization between Dome-GFP and ubiquitin in the wild-type cells (Fig. 5L-L″). In the epidermal cells of *drn^2^* homozygotes, Dome-GFP was markedly higher than that in wild-type embryos in all cases, as observed in images of Dome-GFP obtained at the same gain of signal amplification (Fig. 5B). In images obtained using reduced gain of signal detection, Dome-GFP was observed as larger clumps located near the plasma membrane (Fig. 5B′,B″). To analyze the nature of these clumps, we co-stained Dome-GFP with markers of representative intracellular compartments, including Pdi (endoplasmic reticulum), GM130 (*cis*-Golgi), peanut agglutinin (PNA; *trans*-Golgi), Sec5 (exocytic vesicles), Hrs (early endosomes), Rab5 (early endosomes), Rab7 (late endosomes), Rab11 (recycling endosomes) and LAMP1 (lysosomes), in the epidermal cells of *drn^2^* homozygotes. None of these markers colocalized with Dome-GFP (Fig. 5C-K″). However, large clumps of Dome-GFP often colocalized with ubiquitin (Fig. 5M-M″). Our quantitative analyses revealed that 3.6% of these Dome-GFP clumps colocalized with ubiquitin and 5.3% of ubiquitin-positive vesicles colocalized with Dome-GFP. We speculate that Dome-GFP localizes with ubiquitylated cargos only temporarily because colocalization was observed only in a subset of these vesicles. Nevertheless, as ubiquitylation regulates endosomal trafficking and sorting, these results suggest that Dome-GFP accumulates in atypical endosomal compartments in *drn* mutants. Such a defect in endocytosis may cause JAK/STAT signaling to deteriorate, supporting previous suggestions that endocytosis is essential for Dome activation (Devergne et al., 2007; Moore et al., 2020). Additionally, defective endocytosis may prevent the degradation of Dome-GFP in lysosomes of *drn* mutants. Unlike Dome-GFP, the Wnt and Notch signaling receptors Frizzled 2 (Fz2) and Notch, respectively, were unchanged in the epidermis of wild-type and *drn^2^* homozygotes (Fig. S5). Hence, defective endocytosis in *drn^2^* homozygotes is specific to Dome-GFP.

**Fig. 6.**
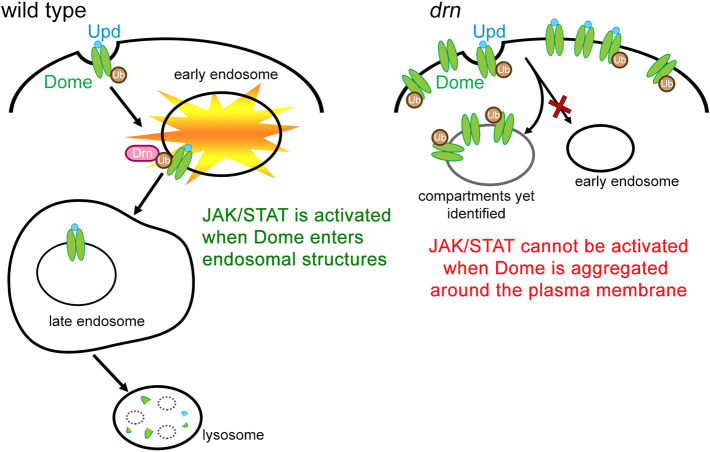
**Drn is required for Dome endocytosis, which contributes to the activation of JAK/STAT signaling.** Schematic showing the potential role of Drn in the endocytic trafficking of Dome. In the presence of Drn, ubiquitylated Dome is transported to late endosomes through the endocytic pathway. The interaction of Drn with ubiquitylated Dome may be required for the internalization of Dome, a process that enables the activation of Dome and STAT. Such internalization is also required for the proper degradation of Dome in lysosomes. In contrast, in the absence of Drn, Dome fails to properly proceed in the endocytic trafficking pathway, which attenuates JAK/STAT signaling activity. Moreover, the failure of endocytosis leads to the abnormal accumulation of Dome in atypical endocytic compartments containing ubiquitin, which are yet to be identified.

Considering the potential binding of Drn to ubiquitin, we determined whether Dome-GFP and Drn could colocalize in wild-type cells. Although the staining patterns of Drn and Dome-GFP did not broadly resemble each other, we found that Drn and Dome-GFP often colocalized with each other (white arrowheads in Fig. 5N-N″). Our quantitative analyses revealed that 3.5% of Dome-GFP colocalized with Drn and 5.8% of Drn colocalized with Dome-GFP. These results suggest that Drn interacts with the ubiquitin moiety on Dome in some endocytic compartments to facilitate proper Dome trafficking. However, such an interaction may be transient because vesicles demonstrating colocalization did not account for a majority. Nevertheless, based on these results, we speculated that ubiquitylated Dome can be recognized by Drn, which can specifically promote the endocytic transportation and degradation of Dome. Dome was accumulated in the atypical endocytic compartment with ubiquitylated cargoes and JAK/STAT signaling was attenuated in *drn* mutants, indicating that such an endocytic process is crucial for activating JAK/STAT signaling (Fig. 6).

## DISCUSSION

### Drn contributes to the endocytic trafficking of Dome, which may be coupled with the activation of JAK/STAT signaling

In this study, we demonstrate that *drn*, which encodes a *Drosophila* ortholog of AWP1, plays crucial roles in the LR asymmetric development of the FG and AMG by positively regulating JAK/STAT signaling. In this process, wild-type *drn* is required for the normal endocytic trafficking of Dome, which is the *Drosophila* JAK/STAT signaling receptor. Whether the internalization as well as endocytic trafficking of Dome is required to activate JAK/STAT signaling in *Drosophila* has been a topic of debate (Devergne et al., 2007; Moore et al., 2020; Ren et al., 2015; Vidal et al., 2010). Analyses of mutations affecting Dome endocytosis have revealed that Dome must be internalized into early endosomes to activate JAK/STAT signaling (Devergne et al., 2007). In contrast, the RNAi-mediated knockdown of genes required for Dome endocytosis was found to enhance JAK/STAT signaling activity, demonstrating that Dome endocytosis negatively regulates JAK/STAT signaling (Vidal et al., 2010). This discrepancy can be explained by the multiple ramifications and parallelism of the endocytic pathway, as described in recent studies (Cendrowski et al., 2016). In particular, blocking a particular step along the pathway can divert endocytic trafficking into various positive or negative regulatory cascades of cell signaling pathways. The relative contributions of endocytic regulators can also change the course of endocytic trafficking, as is well documented in Notch signaling (Shimizu et al., 2014). Moreover, a change in the balance of regulators may change how endocytosis regulates cell signaling pathways according to the context. Hence, to understand comprehensively how endocytosis contributes to the activation of Dome, it is necessary to examine the point at which endocytosis is disrupted and the altered course of endocytic trafficking when normal endocytosis fails.

A study using RNAi of cultured *Drosophila* cells identified *drn* as a negative regulator of JAK/STAT signaling; this finding appears to be opposite to our model (Vidal et al., 2010). However, as other genes required for Dome endocytosis were also identified as negative regulators of JAK/STAT signaling in that analysis, it is likely that this discordance regarding the role of Drn in JAK/STAT signaling results from a discrepancy in the contribution of Dome endocytosis to JAK/STAT signaling, as discussed above (Cendrowski et al., 2016). Regarding this discrepancy, it has been proposed that long-term, loss-of-function analyses, including analyses of mutants in which Dome endocytosis is disrupted, may reflect unexpected cell fate changes induced by altered endocytic pathways and their influence on JAK/STAT signaling (Vidal et al., 2010). However, in this study, we examined three developmental contexts in which JAK/STAT signaling is reduced in *drn* mutants. One of these involves the regulation of *eve* expression, which occurs before cell fate specification and during a short period in early embryogenesis; thus, it does not fit well with a mechanism involving unexpected cell fate changes. Therefore, we propose a model in which Drn is required in some steps of the endocytic trafficking of Dome, which plays a role in the subsequent activation of JAK/STAT signaling (Fig. 6). Our model is consistent with that proposed by Devergne et al., in which the endocytic trafficking of Dome activates JAK/STAT signaling and also provides a mechanism to regulate it quantitatively (Devergne et al., 2007). However, as where and how Drn can control the endocytic trafficking of Dome remain unclear, the coincidence between previous and current results should be interpreted with caution.

### Drn is specifically involved in the endocytic trafficking of Dome

Drn is the *Drosophila* ortholog of AWP1, which binds to ubiquitin and modulates the functions of ubiquitylated proteins in mammals and *Xenopus* species (Chang et al., 2011; Duan et al., 2000; Fenner et al., 2009; Miyata et al., 2012; Seo et al., 2013). It is known that ubiquitylation of the receptors involved in JAK/STAT signaling is important for regulating signaling activities in mammals (Gesbert et al., 2005; Martinez-Moczygemba et al., 2007; Wölfler et al., 2009). The sorting of ubiquitylated membrane proteins into intraluminal vesicles relies on protein complexes in the ESCRT family (Babst et al., 2002a,b; Katzmann et al., 2001). ESCRT-0, ESCRT-I, and ESCRT-II include multiple ubiquitin-binding proteins and interpret ubiquitin as a signal to sort membrane proteins (Clague et al., 2012). In *Drosophila* mutants of the ESCRT-0 complex components *Hrs* and *Stam*, ubiquitylated membrane proteins, such as Notch and Dome, aggregate at the cell cortex and in intracellular compartments (Jékely and Rørth, 2003; Tognon et al., 2014). As such intracellular compartments, including aggregated Dome, were stained with an anti-ubiquitin antibody, it has been proposed that *Drosophila* Dome is ubiquitylated and its endosomal sorting is controlled by ubiquitylation; however, the ubiquitylation of Dome has not been confirmed biochemically (Tognon et al., 2014). We found that a loss of Drn caused Dome to accumulate in large clumps that frequently colocalized with ubiquitin but were not labeled by markers of typical intracellular compartments (Fig. 5C-K″). In addition, Drn occasionally colocalized with markers of various endocytic compartments, demonstrating that Drn is an endocytic protein (Fig. 4). Hence, we speculate that Drn plays a role in the ubiquitin-dependent internalization or sorting of Dome through its potential ubiquitin-binding activity (Fig. 6). This idea is consistent with our observation that Drn often colocalizes with Dome in some intracellular vesicles in wild-type *Drosophila* (Fig. 5N-N″). Thus, in our model, Dome may be misrouted to atypical endocytic compartments, where it fails to be phosphorylated in the absence of Drn. Conversely, differences in Dome trafficking routes between wild-type and *drn* mutant embryos should help identify the endocytic compartment where Dome is activated by phosphorylation. However, it is difficult to delineate incorrect Dome trafficking routes in the *drn* mutant because Dome did not specifically colocalize with typical markers of endocytic compartments under this condition. Our model also predicts that such misrouting can consequently prevent the degradation of Dome in lysosomes and leave it to accumulate, as observed in *drn* mutants (Fig. 6).

Our analyses revealed that *drn* mutations induce marked accumulation of Dome but not of Notch or Fz2 (Fig. S5). The intracellular distribution of Notch and Fz2 in the *drn* mutant appeared to be similar to that in the wild type. Thus, Drn is not a general component of endosomal protein sorting but is specific to Dome; however, how such specificity is achieved remains unclear.

### Roles of JAK/STAT signaling in LR asymmetric development of the *Drosophila* gut

In this study, we found that JAK/STAT signaling activity must be maintained at proper levels for normal LR asymmetric development of the FG and AMG. We previously observed a similar phenomenon in Wnt or JNK signaling activity (Kuroda et al., 2012; Taniguchi et al., 2007). However, we failed to detect any LR asymmetry in the activity or the distribution of molecules involved in the JAK/STAT, Wnt and JNK signaling pathways (Fig. S6) (Kuroda et al., 2012; Taniguchi et al., 2007). As these three signaling pathways are required for LR asymmetric rearrangement of nuclei in the visceral muscles of the MG, they may play permissive roles in rearranging these nuclei upon a common cue, which is yet unknown, of LR polarity.

AWP1/Drn is highly conserved from *Drosophila* to humans (Fig. 1E). As receptors in the mammalian JAK/STAT pathway are also ubiquitylated, we suggest that the role of AWP1/Drn in JAK/STAT signaling and LR asymmetric development is evolutionarily conserved in various organisms (Gesbert et al., 2005; Martinez-Moczygemba et al., 2007; Wölfler et al., 2009).

## MATERIALS AND METHODS

### Fly stocks

We used Canton-S as the wild-type *Drosophila* strain. We generated the *drn^1^* and *drn^2^* mutants in this study. *drn^GS12294^* and *drn^GS10567^* are previously reported GS lines (Toba et al., 1998). We also generated *UAS-drn* and *UAS-myc-drn* in this study. *UAS-hop^Tuml^* (Harrison et al., 1995) and *UAS-dome-GFP* (Ghiglione et al., 2002) have been previously described. *UAS-drnRNAi* (Vienna *Drosophila* Resource Center, #103508) was used for RNAi against *drn*. The following GAL4 lines were used in this study: *da-GAL4* (Wodarz et al., 1995), *48Y-GAL4* (Martin-Bermudo et al., 1997), *24B-GAL4* (Brand and Perrimon, 1993), *hand-GAL4* (Popichenko et al., 2007), *elav-GAL4* (Yao and White, 1994), *NP1522* (Hayashi et al., 2002), *NP5021* (Hayashi et al., 2002), *NP0221* (Hayashi et al., 2002) and *65E04* (Jenett et al., 2012). The lines used to generate homozygotes of *drn^2^* lacking its maternal contribution were *P{ry^+t7.2^=neoFRT}82B ry^506^* (Bloomington *Drosophila* Stock Center, #2035) and w*;P{ry^+t7.2^=neoFRT}82B P{w^+mC^=ovoD1-18}3R/st^1^βTub85D^D^ss^1^e^s^/TM3, Sb^1^ (*Drosophila* Genomics Resource Center, #106675).

All fly stocks were maintained on standard *Drosophila* medium at 25°C, unless stated otherwise. Mutant alleles of the second and third chromosomes were balanced with appropriate blue balancers, such as *CyO, P{en1}wg^en11^*, *TM6B, AbdA-lacZ* and *TM3, ftz-lacZ*.

### Generation of *drn^1^* and *drn^2^* mutants

We generated the *drn*-deletion mutants *drn^1^* and *drn^2^* by the imprecise excision of P elements from *GS12689* and *GS10487*, respectively (Toba et al., 1998). Imprecise excision was performed using a standard procedure described previously (Hummel and Klämbt, 2008). *drn^1^* and *drn^2^* mutations contain deletions from 9,353,341 to 9,355,167 and from 9,353,293 to 9,358,171, respectively (FlyBase2015_03, Dmel Release 6.06).

### Generation of homozygotes for *drn* lacking maternal contribution

We obtained *drn^2^* homozygous embryos lacking the *drn* maternal contribution (*drn^2 m/z^*) using standard crosses, as described previously (Prudencio and Guilgur, 2015). In brief, *FRT82B* was introduced into the *drn^2^* chromosome by recombination. Flies carrying *FRT82B drn^2^* were selected by Geneticin (Gibco) and genomic PCR using the primers 5′-TCACGCATTCAGAGCTTCGTGTGCCC-3′ and 5′-ATGTTGCTGCGTTTGCTCTGCGTATTCCAC-3′. *FRT82B drn^2^/TM3b, Sb* females were crossed with *hsFLP/Y; FRT82B ovoD/TM3, Sb* males to obtain *hsFLP/+; FRT82B ovoD/FRT82B drn^2^* females through heat-shock treatment. These females were then crossed with *FRT82B drn^2^/TM3b, Sb* males to obtain *FRT82B drn^2^* homozygous embryos without the *drn* maternal contribution.

### Generation of UAS*-drn* and UAS*-myc-drn* transgenic flies

To construct *UAS-drn*, a cDNA fragment composed of an entire open reading frame of a *drn* transcript (CG45050-RC) was PCR amplified using an upper strand primer containing an EcoRI site (5′-CCGGAATTCAGCAGGAAGCAGACGAAACT-3′) and a lower-strand primer containing HindIII and BglII sites (5′-CCCCAAGCTTAGATCTTCCTTGTTATAGCGCAGCAT-3′). The cDNA clone RE70963 was used as a template (Stapleton et al., 2002). The PCR product was digested with EcoRI and HindIII, subcloned into the EcoRI and HindIII sites of pBluescript, and sequenced (Agilent Technologies). The cloned fragment was subcloned into *the EcoRI and BglII* sites of the pUAST vector (Brand and Perrimon, 1993).

To construct UAS-*myc-drn*, a DNA fragment composed of an entire open reading frame of a *drn* transcript (CG45050-RC) was PCR amplified using RE70963 cDNA as a template, an upper strand primer containing EcoRI and BglII sites and the *myc*-tag coding sequence (underlined) (5′-CCGGAATTCCAAAATGGAGCAGAAGCTGATCTCGGAGGAGGATCTGAGATCTATGGAACGTGAATCTAACCC-3′), and a lower strand primer containing a XhoI site (5′-CCGCTCGAGTCAAATCTTTTGAATCTTCT-3′). CG45050-RC has the same open reading frame as CG45050-RB, -RD, -RE, -RF and -RG (Fig. 1D). The PCR product was digested with EcoRI and XhoI and subcloned into pUAST. The DNA sequence of the coding region was then confirmed. UAS-*drn* and UAS-*myc-drn* constructs were introduced into the *Drosophila* genome using P element-mediated transformation (Spradling and Rubin, 1982).

### Generation of the anti-Drn antibody

A fragment of *drn* cDNA (RE70963) containing an entire open reading frame of CG45050-RC was amplified by PCR, sequenced, and subcloned into the BamHI and EcoRI sites of the pGEX-2T vector (GE Healthcare Life Sciences). A GST-Drn fusion protein was produced in Origami B (DE3) cells (Novagen) and purified using a Glutathione Sepharose 4B column. The purified GST-Drn fusion protein was used to immunize rats, and polyclonal antiserum was purified using a standard protocol.

### Antibody staining, *in situ* hybridization and microscopic analysis

Embryos were immunostained as described previously using the following primary antibodies: mouse anti-Fas3 [1:100, Developmental Studies Hybridoma Bank (DSHB), 7G10], chicken anti-β-galactosidase (1:500, Abcam, ab9361), mouse anti-Pdi (1:200, Stressgen, 1D3), anti-lectin-PNA (1:500, Vector Laboratories), mouse anti-Sec5 (1:200, 22A2; Murthy et al., 2003), rabbit anti-GM130 (1:50, Abcam, ab30637), guinea pig anti-Rab5 (1:3000, gift from Akira Nakamura, Kumamoto University, Japan), rabbit anti-Rab7 (1:5000; Tanaka and Nakamura, 2008), rabbit anti-Rab11 (1:5000; Tanaka and Nakamura, 2008), guinea pig anti-Hrs (1:1000; Lloyd et al., 2002), mouse anti-multiubiquitin antibody (1:200, MBL, FK2), rabbit anti-LAMP1 antibody (1:1000, Abcam, ab30687), mouse anti-extra domain of Notch (1:500, DSHB, C458.2H), mouse anti-Frizzled 2 (1:20, DSHB, 1A3G4), rabbit anti-GFP (1:500, MBL, 598), rabbit anti-RFP (1:500, MBL, PM005), rat anti-GFP (1:500, Nacalai Tesque, 04404-26), mouse anti-Eve (1:20, DSHB, 3C10) and rat anti-Drn (1:500). The chitin-binding probe CBP546 was prepared from a bacterial expression construct using the protocol provided by Yinhua Zhang (New England Biolabs) (Dong et al., 2014). CBP546 (1:50) was added along with secondary antibodies for other primary antibodies to visualize the trachea. Images were generated using an LSM880 (Carl Zeiss) microscope and processed using Adobe Photoshop. For *in situ* hybridization, standard protocols were used, as described previously (Jiang et al., 1991). Images were obtained using an Axiosop2 Plus microscope (Carl Zeiss).

### Quantitative analyses of colocalizations in intracellular vesicles

The same threshold was set for each pair of images (two channels representing respective markers) using ImageJ. Using the ‘Analyze Particles’ function, particles larger than five-pixel units were defined as the region of interest (Roi). Roi sets from two channels of an image were compared. Each Roi overlapping between two channels was defined as a particle demonstrating colocalization and was used for calculating the colocalization rate between two markers. The number of Rois showing colocalization against the total number of Rois was calculated as the percentage of colocalization.

## Supplementary Material

10.1242/develop.201224_sup1 Supplementary information
