# Supplementary material for: The Drosophila AWP1 ortholog Doctor No regulates JAK/STAT signaling for left–right asymmetry in the gut by promoting receptor endocytosis
Source: Development. 2023 Mar 21;150(6):dev201224. doi: 10.1242/dev.201224 (PMC10112927; doi:10.1242/dev.201224)
Supplement: Supplementary information [file develop-150-201224-s1.pdf]

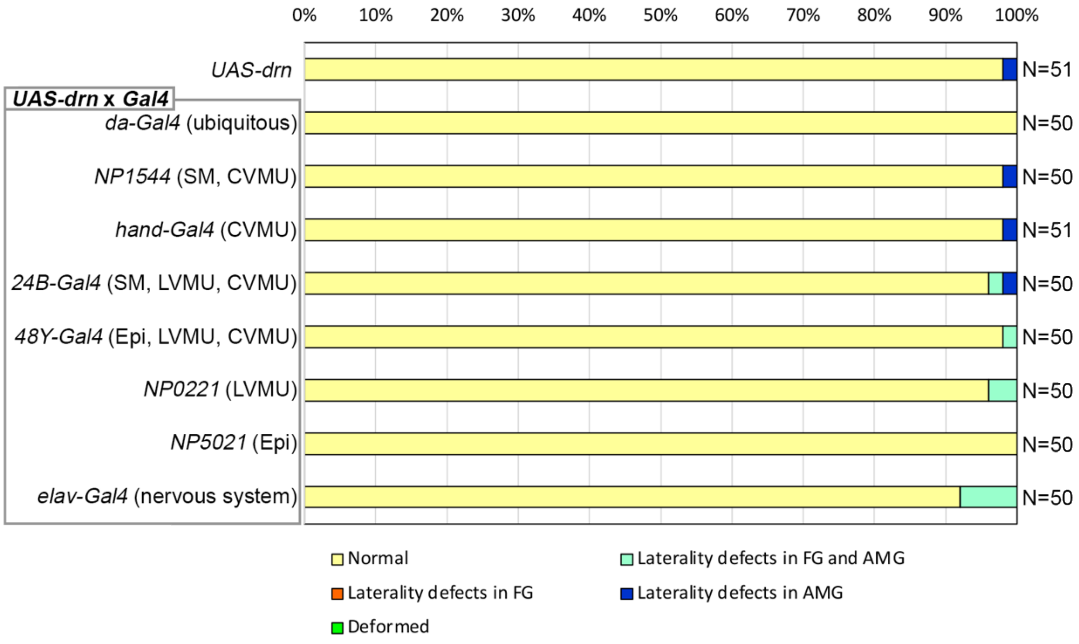

Fig. S1. *drn* misexpression in various tissues of wild-type embryos has little effect on FG or AMG LR asymmetry.

The frequency of LR asymmetry defects (%) in the FG and AMG of embryos carrying *UAS-drn* without any GAL4 driver (control, *UAS-drn*) or with the respective GAL4 drivers shown at the left. Bars denote the percentage of embryos with no laterality defects (yellow), with laterality defects in only the FG (orange) or the AMG (blue), or in both the FG and AMG (turquoise), or with deformities (green). The number (N) of embryos scored is shown on the right.

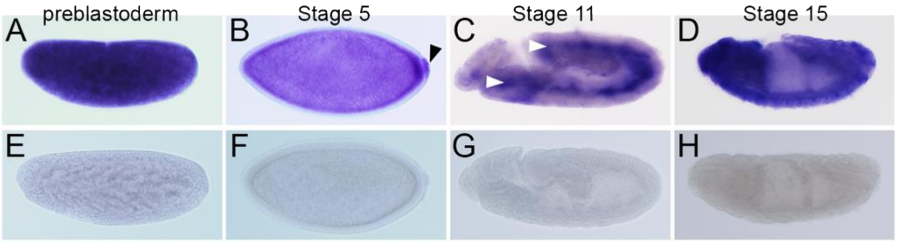

Fig. S2. *drn* expression is detected by *in situ* hybridization at various embryonic stages. (A–H) Whole-mount *in situ* hybridization with anti-sense (A–D) and sense RNA probes (E–H) against *drn* at the preblastoderm stage (A, E), stage 5 (B, F), stage 11 (C, G), and stage 15 (D, H) of wild-type embryos. The black arrowhead in B indicates pole cells. White arrowheads in C indicate the TVM.

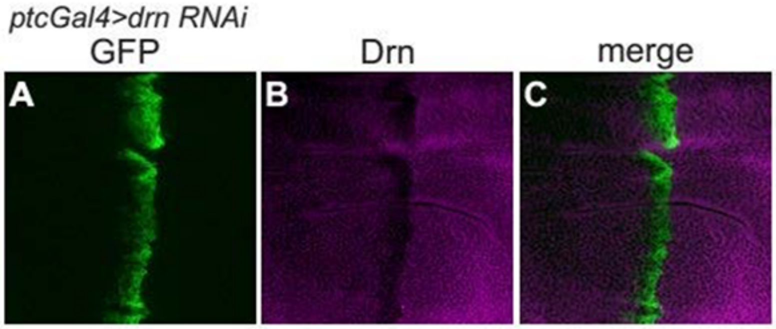

Fig. S3. Anti-Drn antibody specifically detects endogenous Drn.
(A–C) In the wing discs of third-instar larvae, *drn* gene was knocked down by RNAi under the control of *ptc-GAL4*, where *UAS-GFP* was expressed (green in A, C). These wing discs were stained with anti-Drn antibody (magenta in B, C). C shows a merged image of A and B.

Development • Supplementary information

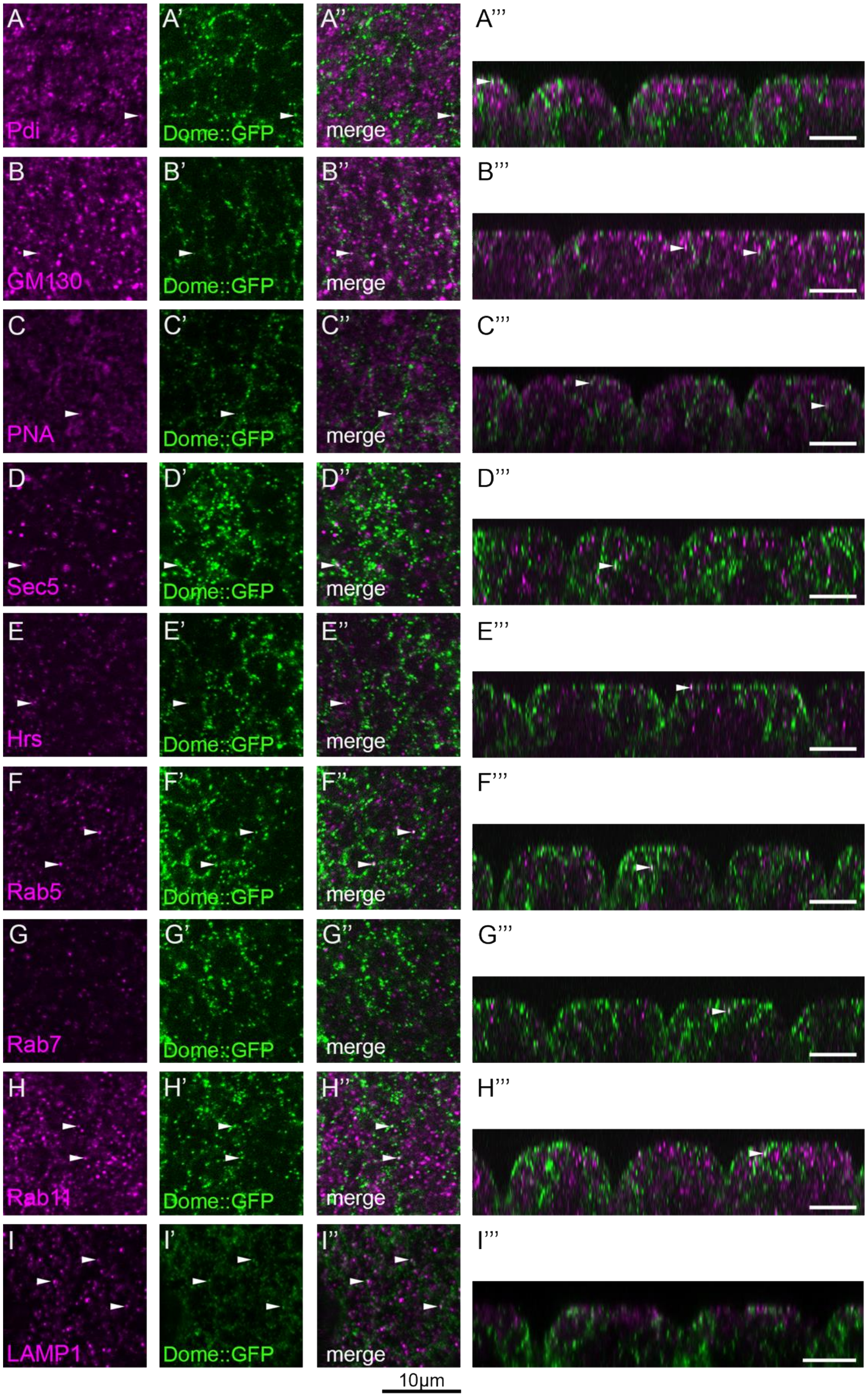

A
Pdi
A'
Dome::GFP
A''
merge
A'''
B
GM130
B'
Dome::GFP
B''
merge
B'''
C
PNA
C'
Dome::GFP
C''
merge
C'''
D
Sec5
D'
Dome::GFP
D''
merge
D'''
E
Hrs
E'
Dome::GFP
E''
merge
E'''
F
Rab5
F'
Dome::GFP
F''
merge
F'''
G
Rab7
G'
Dome::GFP
G''
merge
G'''
H
Rab11
H'
Dome::GFP
H''
merge
H'''
I
LAMP1
I'
Dome::GFP
I''
merge
I'''
10μm

Fig. S4. Dome occasionally colocalizes with markers of various endocytic compartments in the epidermis of wild-type embryos.

(A–I''') Dome subcellular localization in the epidermal cells of wild-type embryos. Embryos were double stained with anti-GFP antibody (green, middle and right columns) and against the following markers of intracellular compartments (magenta, left and right columns): (A–A''') Pdi (ER), (B–B''') GM130 (*cis*-Golgi), (C–C''') PNA (*trans*-Golgi), (D–D''') Sec5 (exocyst), (E–E''') Hrs (early endosomes), (F–F''') Rab5 (early endosomes), (G–G''') Rab7 (late endosomes), (H–H''') Rab11 (recycling endosomes), and (I–I''') LAMP1 (lysosome). (A''–I'') Merged images of A–I and A'–I', in the given order. A'''–I''' shows sagittal sections from A''–I'', in the given order. White arrowheads indicate vesicles showing the colocalization of Dome with markers of various endocytic compartments. Scale bar: 10 μm.

Development • Supplementary information

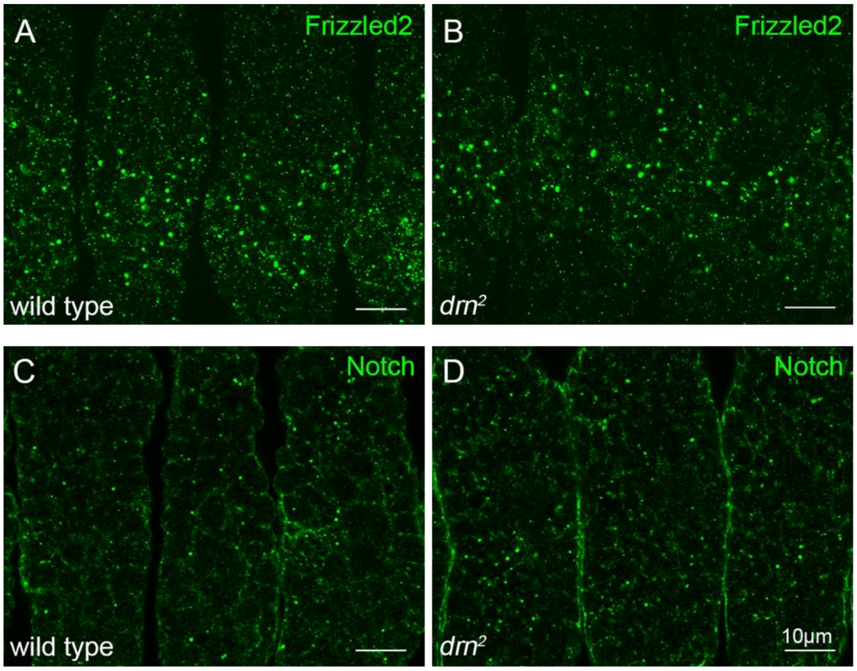

Fig. S5. Drn is not required for proper Fz2 or Notch trafficking.

(A–D) The distribution of Fz2 and Notch, detected by anti-Fz2 (A and B) and anti-Notch (C and D) antibody staining, in the epidermis of wild-type (A, C) or *drn*^{2} homozygous (B, D) embryos. Scale bar: 10 µm.

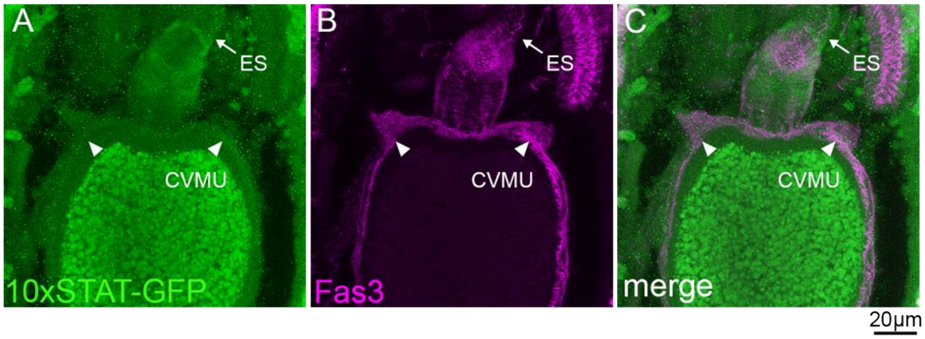

Fig. S6. JAK/STAT signaling activity in the AMG has no obvious LR asymmetry.
(A–C) JAK/STAT signaling in the FG and AMG, detected by the reporter gene *10×STAT-GFP* (green in A, C), at stage 15 in wild-type embryos (ventral view). GFP derived from *10×STAT-GFP* was detected by anti-GFP antibody staining. The visceral muscles overlaying the PV and AMG were detected by anti-Fas3 antibody staining (magenta in B, C). (C) Merged images of panels A and B. Arrows and arrowheads denote the activation of *10×STAT-GFP* in the ES and CVMU, respectively. Scale bar: 20 µm.

Development • Supplementary information
