# Supplementary material for: The Drosophila AWP1 ortholog Doctor No regulates JAK/STAT signaling for left–right asymmetry in the gut by promoting receptor endocytosis
Source: Development. 2023 Mar 21;150(6):dev201224. doi: 10.1242/dev.201224 (PMC10112927; doi:10.1242/dev.201224)
Supplement: Supplementary file 1 [file develop-150-201224_review_history.pdf]

The Drosophila AWP1 ortholog Doctor No regulates JAK/STAT signaling for left-right asymmetry in the gut by promoting receptor endocytosis

Yi-Ting Lai, Takeshi Sasamura, Junpei Kuroda, Reo Maeda, Mitsutoshi Nakamura, Ryo Hatori, Tomoki Ishibashi, Kiichiro Taniguchi, Masashi Ooike, Tomohiro Taguchi, Naotaka Nakazawa, Shunya Hozumi, Takashi Okumura, Toshiro Aigaki, Mikiko Inaki and Kenji Matsuno
DOI: 10.1242/dev.201224

Editor: Thomas Lecuit

Review timeline
Original submission: 20 August 2022
Editorial decision: 10 October 2022
First revision received: 23 January 2023
Accepted: 9 February 2023

Original submission

First decision letter

MS ID#: DEVELOP/2022/201224

MS TITLE: The Drosophila AWP1 ortholog Doctor No regulates JAK/STAT signaling for left-right asymmetry in the gut by promoting receptor endocytosis

AUTHORS: Yi-Ting Lai, Takeshi Sasamura, Junpei Kuroda, Reo Maeda, Mitsutoshi Nakamura, Ryo Hatori, Tomoki Ishibashi, Kiichiro Taniguchi, Masashi Ooike, Tomohiro Taguchi, Naotaka Nakazawa, Shunya Hozumi, Takashi Okumura, Toshiro Aigaki, Mikiko Inaki, and Kenji Matsuno

I have now received all the referees reports on the above manuscript, and have reached a decision. The referees' comments are appended below, or you can access them online: please go to BenchPress and click on the 'Manuscripts with Decisions' queue in the Author Area.

The overall evaluation is positive and we would like to publish a revised manuscript in Development, provided that the referees' comments can be satisfactorily addressed. In particular one referee, who is more critical of the paper, requests a better characterization/substantiation of the claimed endocytosis defects. Please attend to all of the reviewers' comments in your revised manuscript and detail them in your point-by-point response. If you do not agree with any of their criticisms or suggestions explain clearly why this is so. If it would be helpful, you are welcome to contact us to discuss your revision in greater detail. Please send us a point-by-point response indicating your plans for addressing the referee’s comments, and we will look over this and provide further guidance.

Reviewer 1

Advance summary and potential significance to field

In this article, thanks to an elegant genetic screen, the authors report the identification of the ubiquitin-binding protein Doctor no (Drn)/AWP1 as a regulator of left-rigth asymmetric gut morphogenesis. By tissue-specific rescue experiment, they show that Drn is required in the circular

© 2023. Published by The Company of Biologists under the terms of the Creative Commons Attribution License (https://creativecommons.org/licenses/by/4.0/).

visceral muscle (CVMU) cells. Based on their previous findings and on literature (Vidal et al, 2010), they next test the possibility that Drn regulates JAK/STAT signaling pathway. The convincingly show that mutations of several genes involved in the JAK/STAT signaling pathway impairs LR asymmetry in a similar manner as does the loss of Drn. Having establish that the level of JAK/STAT signaling is key for the asymmetric development of the anterior gut, they nicely show that Drn is required for the proper expression of even-skipped and for trachea morphogenesis, indicating that Drn is likely a general component of the JAK/STAT signaling pathway. With the exception of the relationship between Stat92E and Drn (see my comment below), the first part of the manuscript is convincing. In the second part, the authors aimed to decrypt the mechanisms by which Drn could regulate JAK/STAT signaling through a series of localization studies. Because of tissue constrains limiting the ability to image cells of interest that are located too deeply in the embryo, the authors analyze the localization in epidermal cells located on the surface. Although I understand the methodological bottleneck, this represent a strong limitation in my opinion. The only clear result of this second section concerns the increased immunostaining of overexpressed Domeless-GFP in drn2 mutant epidermal cells, indicating that the protein levels of Dome depend on Drn. Using a series of intracellular markers, they did not find prominent colocalization of Drn and Dome with endosomal markers. They did not design an experiment to demonstrate that Dome endocytosis is affected, an experiment required to support their claim. It seems rather that Dome is accumulated in an unidentified intracellular compartment in drn mutant, a finding that may argue against a blockage of receptor endocytosis.

Despite its potential interest, based on these major limitations on the cell biology part of the study, I believe that the study is still at a too preliminary and inconclusive stage to be accepted for publication in Development.

Comments for the author

Main points:
- Contrary to what is stated in the title and all along the manuscript, the authors do not provide evidence for a role of Drn in Domeless endocytosis.
The authors found and report that overexpressed Domeless-GFP accumulates in clumps and yet to be identified intracellular compartment upon loss of Drn. It is not determined whether Domeless that accumulates intracellularly comes from the secretory or endocytic pathway? A Domeless internalization assay would be needed to address this issue.
Based on the data, I feel that the fact that Dome accumulates intracellularly argues against a role for Drn in endocytosis. It is also unclear whether Dome is a target of Drn, the colocalization between Drn and Dome appears to be low and is not quantified.

- Did the authors consider staining drn mutant tissues with anti-(mono and poly)Ubi antibodies. The reason I ask is that assuming that the Domeless is ubiquitinated (a fact that, contrary to what the authors claim, is not directly demonstrated in the reference Tognon et al, 2014), and that Drn selectively recognizes the Ubi moiety on Domeless, one prediction is that the accumulated Domeless in drn mutant is ubiquitinated and cannot be processed further due to the lack of Drn activity. Along the same vein, it is not clear if Domeless is a direct target of Drn, this would require the mapping of ubiquitination site(s) on Domeless, determine its ability to bind to Drn…

- The localization studies were performed in epidermal cells overexpressing Domeless-GFP, not cells in which Drn physiologically regulates LR asymmetry. Although I fully understand that based on the accessibility of the tissue , the imaging of cells of interest is difficult, I am questioning the conclusions that can be drawn/biological relevance of the observations. Are the regulators of JAK/STAT pathway and membrane trafficking regulators, potential Dome-specific E3 ligase,… expressed similarly in epidermal cells than in gut cells ? along the same line, the authors raise the apparent discrepancies between the physiological, in vivo, positive role of endocytic pathway in the regulation of JAK-STAT signaling in Drosophila egg chamber (Devergne et al, 2007) and the negative role of endocytosis obtained in vitro in KC167 cells (Vidal et al, 2010). As a developmental cell biologist, I tend to give more weight to the physiological context in vivo than to the cultured cells.

- The schematical representation depicted in Fig. 6 is inappropriated in my opinion: what are the evidence that Drn localizes in internal vesicles inside LE (multivesicular body?)? The ubiquitin moiety is removed prior to incorporation into MVBs. There are no evidence that Domeless is

© 2023. Published by The Company of Biologists under the terms of the Creative Commons Attribution License (https://creativecommons.org/licenses/by/4.0/).

Ubiquitinated in the absence of Drn? And there no evidence that it is the internalized Domeless-GFP that accumulates in the ‘unknown compartments’?

Minor comment
- Figure 1G: difference between AMG defect (middle) and FG + AMG defect (right panel) is difficult to see/apprehend for non-expert, schematical representations would help.

- Figure 3K: the rescue of Stat92E 06346 mutant phenotype by misexpression of drn is interesting. It would be worthwhile to elaborate on how the authors envisage that Drn can rescue Stat92E in molecular terms.
The term epistasis is employed. To my knowledge, this term can be used when testing two mutants genes, not for misexpression of a gene.
Perhaps to test for epistasis, analyzing the double mutant context, drn together with dome/upd/Stat92E or hop will be informative? One prediction is that for two genes involved in the same pathway, in case of null genetic alleles, the phenotype of the double mutant should be non-additive.

<u>Reviewer 2</u>

Advance summary and potential significance to field

Drosophila exhibits laterality in the anterior and posterior regions of the gut. However, the both regions are regulated by different genetic pathways. While the genetic and cellular mechanism underlying the laterality of posterior region of the gut has been studied in detail, the anterior region of the gut remain unexplored. In this paper, the authors have performed a genetic screen and have searched for mutants defective in the laterality of the anterior gut. They have identified two mutants, both of which are mapped to a single gene drn. Genetic data suggest that Drn is a component of JAK/STAT signaling required for endocytic trafficking of the ligand Dome. In support of this, Dome was accumulated in the epidermis of drn mutant, which show additional phenotypes such as aberrant expression of even-skipped and abnormal trachea branching, typical of JACK/STAT signaling defects. I believe that the paper provides two lines of new information for our understanding of laterality in Drosophila and of JACK/STAT signaling pathway, and these findings deserve publication in Development.

Comments for the author

I only have minor comments.

1) The authors may speculate why only a portion (~30%) of drn mutants show laterality defects in AMG and/or FG (for example, drn2 in Fig. 1F).

2) I believe that the image shown in Fig. 5B” is lower gain than its counterpart A’. This should be mentioned clearly in the legend to avoid misunderstanding.

3) Dome protein was accumulated in the epidermis of drn2 mutant. Were Dome accumulates localized in lysosome as speculated by the authors? It would be nice to see co-staining with LAMP1 in Fig. 5.

4) Drn protein localization was studied in the epidermis in Fig. 4 and Fig. 5. I wonder if Drn is similarly localized in the anterior gut cells.

<u>Reviewer 3</u>

Advance summary and potential significance to field

In their manuscript, Lai et al. identify the conserved ubiquitin-binding protein AWP1 ortholog as an important regulator of left-right asymmetry of the foregut in Drosophila. They further show that drn is a positive regulator of the Jak/Stat signaling pathway, proposing that drn controls

© 2023. Published by The Company of Biologists under the terms of the Creative Commons Attribution License (https://creativecommons.org/licenses/by/4.0/).

internalization, traffic and signaling of the Dome receptor in this process. These are interesting and new results, allowing a better understanding of how the foregut acquires its asymmetry through the activity and endocytic control of the Jak/Stat pathway.

I have only relatively minor comments that may improve clarity and strengthen some results.

- for clarity, please mark Left and Right on pictures and graphs

- results p3 line21 : ‘located on the right side of the midline’, do the authors mean ‘left side’ (fig1A) ?

- the drn2 excision allele is proposed to be a null mutation, however, it has a weaker phenotype than the original P insertion allele (fig1F)

- it is not completely clear what the authors call a ‘defect in AMG’ (p. 4). Could you please clarify ?

- it would be a nice confirmation if authors could recapitulate the phenotype driving drn-RNAi in CVMU cells

- p5 , first paragraph: have the authors tried to invalidate Jak/Stat activity in CVMU cells using RNAi ?

- fig3F,F’ : embryos do not seem to be properly oriented along the DV axis

- p6, drn-antibodies : did the authors tested antibody reactivity upon drn overexpression?

- trafficking (figs4&5): this part is missing quantification to make a strong point regarding Drn localization in endocytic compartments. Also, correlation coefficients (e.g., Pearson’s and Manders) should be used to confirm co-localization

- Introduction line 11: Speder et al 2006 should be acknowledged when refering to the identification of myo31DF/myo1D. As for nomenclature, myo1D should be prefered as it is now the standard name for the gene in both flies and vertebrates

- Introduction p3 last line : Devergne et al 2002 should be acknowledged when refering to Dome identification

- Introduction line 18 : Ligoxygakis et al, should not be cited as its conclusions (maternal origin of gut LR asymmetry) have been clearly and elegantly refuted by Hayashi et al. 2005

Comments for the author

In their manuscript, Lai et al. identify the conserved ubiquitin-binding protein AWP1 ortholog as an important regulator of left-right asymmetry of the foregut in Drosophila. They further show that drn is a positive regulator of the Jak/Stat signaling pathway, proposing that drn controls internalization, traffic and signaling of the Dome receptor in this process. These are interesting and new results, allowing a better understanding of how the foregut acquires its asymmetry through the activity and endocytic control of the Jak/Stat pathway.

I have only relatively minor comments that may improve clarity and strengthen some results.

- for clarity, please mark Left and Right on pictures and graphs

- results p3 line21 : ‘located on the right side of the midline’, do the authors mean ‘left side’ (fig1A) ?

- the drn2 excision allele is proposed to be a null mutation, however, it has a weaker phenotype than the original P insertion allele (fig1F)

- it is not completely clear what the authors call a ‘defect in AMG’ (p. 4). Could you please clarify ?

© 2023. Published by The Company of Biologists under the terms of the Creative Commons Attribution License (https://creativecommons.org/licenses/by/4.0/).

- it would be a nice confirmation if authors could recapitulate the phenotype driving drn-RNAi in CVMU cells

- p5 , first paragraph: have the authors tried to invalidate Jak/Stat activity in CVMU cells using RNAi ?

- fig3F,F' : embryos do not seem to be properly oriented along the DV axis

- p6, drn-antibodies : did the authors tested antibody reactivity upon drn overexpression?

- trafficking (figs4&5): this part is missing quantification to make a strong point regarding Drn localization in endocytic compartments. Also, correlation coefficients (e.g., Pearson's and Manders) should be used to confirm co-localization

- Introduction line 11: Speder et al 2006 should be acknowledged when refering to the identification of myo31DF/myo1D. As for nomenclature, myo1D should be prefered as it is now the standard name for the gene in both flies and vertebrates

- Introduction p3 last line : Devergne et al 2002 should be acknowledged when refering to Dome identification

- Introduction line 18 : Ligoxygakis et al, should not be cited as its conclusions (maternal origin of gut LR asymmetry) have been clearly and elegantly refuted by Hayashi et al. 2005

First revision

Author response to reviewers' comments

Response to Reviewer Comments

Responses to Reviewer 1

Main points:

1) Contrary to what is stated in the title and all along the manuscript, the authors do not provide evidence for a role of Drn in Domeless endocytosis.
The authors found and report that overexpressed Domeless-GFP accumulates in clumps and yet to be identified intracellular compartment upon loss of Drn. It is not determined whether Domeless that accumulates intracellularly comes from the secretory or endocytic pathway? A Domeless internalization assay would be needed to address this issue.
Based on the data, I feel that the fact that Dome accumulates intracellularly argues against a role for Drn in endocytosis. It is also unclear whether Dome is a target of Drn, the colocalization between Drn and Dome appears to be low and is not quantified.

Response:
Thank you very much for your valuable suggestions. Although additional experimentation was requested to reinforce our conclusion, such as an internalization assay, these experiments would be very difficult to conduct. In particular, a specific antibody against the extracellular domain of Dome or a transgenic line of *Dome* encoding Dome with an extracellular tag is not yet available. Therefore, although the experiments suggested by the reviewer could be informative, these experiments could not be included in the revised manuscript.
We reported that Dome occasionally colocalized with various intracellular compartments in the wild type. On the other hand, Dome was accumulated in some clumps that did not overlap with the endoplasmic reticulums (ERs) or Golgi, where Dome is presumably stacked and accumulated under exocytosis disruption. More importantly, as suggested by the reviewer in comment (2), we performed anti-ubiquitin antibody staining (as mentioned in our revised manuscript) and found that the clumps of Dome were often stained with this antibody in the *drn* mutant. This observation supported our idea that Dome is ubiquitinated during endocytosis

© 2023. Published by The Company of Biologists under the terms of the Creative Commons Attribution License (https://creativecommons.org/licenses/by/4.0/).

and that ubiquitination-dependent sorting and/or degradation of Dome does not properly occur in the *drn* mutant [please see our response to comment (2)]. We appreciate this excellent suggestion. Therefore, we believe that these observations serve as strong evidence to support our idea that *drn* is required for the endocytosis of Dome.

We also reported that Dome colocalizes with Drn, suggesting that Dome is a target of Drn. However, as pointed out by the reviewer, the frequency of such colocalization was not quantified in our previous manuscript. As suggested by the reviewer, we have now quantified the percentage of clumps showing colocalization between Drn and Dome in the revised manuscript. Thank you very much for your valuable advice to improve our manuscript. We found that approximately 5.8% of Dome-positive vesicles also contained Drn. Therefore, the frequency of their colocalization was not extremely low, although vesicles demonstrating such colocalization did not account for a large percentage. We hence speculated that such an interaction may be transient; the same has been discussed in the revised manuscript, as mentioned below. In addition, we found that Dome colocalized with ubiquitin in the *drn* mutant. These new results further support our hypothesis that Drn controls the endocytic trafficking of Dome [please see our response to comment (2)]. To explain the new results regarding the quantitation of colocalization between Dome and Drn in the revised manuscript, we have modified the sentences as follows:

Revised sentences:
Results; Drn is required for endocytic trafficking of the Dome receptor; third paragraph, line 1:
Considering the potential binding of Drn to ubiquitin, we determined whether Dome-GFP and Drn could colocalize in wild-type cells. Although the staining patterns of Drn and Dome-GFP did not broadly resemble each other, we found that Drn and Dome-GFP often colocalized with each other (white arrowheads in Fig. 5N–N”). Our quantitative analyses revealed that 3.5% of Dome-GFP colocalized with Drn and 5.8% of Drn colocalized with Dome-GFP. These results suggest that Drn interacts with the ubiquitin moiety on Dome in some endocytic compartments to facilitate proper Dome trafficking. However, such an interaction may be transient because vesicles demonstrating colocalization did not account for a majority. Nevertheless, based on these results, we speculated that ubiquitinated Dome can be recognized by Drn, which can specifically promote the endocytic transportation and degradation of Dome. Dome was accumulated in the atypical endocytic compartment with ubiquitinated cargoes and JAK/STAT signaling was attenuated in *drn* mutants, indicating that such an endocytic process is crucial for activating JAK/STAT signaling (Fig. 6).

Previous sentences:
Considering that Drn may facilitate the endocytic trafficking of Dome, we considered whether Dome-GFP and Drn might colocalize in some intracellular vesicles. Although the staining patterns of Drn and Dome-GFP did not broadly resemble each other, they colocalized in a small population of vesicles (white arrowheads in Fig. 5K-K”). These results suggest that Drn may directly or indirectly interact with Dome in some endocytic compartments to facilitate proper Dome trafficking. This agrees with the finding that Dome is ubiquitinated (Tognon et al., 2014). Taking these results together, we speculate that, as seen in other organisms, the binding of ubiquitin to Drn specifically promotes the internalization of Dome, which is a critical step for activating JAK/STAT signaling (Fig. 6).

Added sentences:

Materials and Methods; **Quantitative analyses of colocalizations in intracellular vesicles:**
The same threshold was set for each pair of images (two channels representing respective markers) using ImageJ. Using the Analyze Particles function, particles larger than five-pixel units were defied as the region of interest (Roi). Roi sets from two channels of an image were compared. Each Roi overlapping between two channels was defined as a particle demonstrating colocalization and was used for calculating the colocalization rate between two markers. The number of Rois showing colocalization against the total number of Rois was calculated as the percentage of colocalization.

© 2023. Published by The Company of Biologists under the terms of the Creative Commons Attribution License (https://creativecommons.org/licenses/by/4.0/).

2) Did the authors consider staining *drn* mutant tissues with anti-(mono and poly) Ubi antibodies. The reason I ask is that assuming that the Domeless is ubiquitinated (a fact that, contrary to what the authors claim, is not directly demonstrated in the reference Tognon et al, 2014), and that Drn selectively recognizes the Ubi moiety on Domeless, one prediction is that the accumulated Domeless in drn mutant is ubiquitinated and cannot be processed further due to the lack of Drn activity. Along the same vein, it is not clear if Domeless is a direct target of Drn, this would require the mapping of ubiquitination site(s) on Domeless, determine its ability to bind to Drn.

Response 2-1:
We found this idea excellent. As suggested, we examined whether Dome colocalized with mono- and polyubiquitin because ubiquitinated receptors are generally transported to endosomes and destined for degradation in lysosomes. We discovered that large aggregations of Dome were occasionally stained by an anti-ubiquitin antibody that could specifically recognize both mono- and poly-ubiquitin in the *drn* mutant (3.6%). However, such colocalization between Dome and ubiquitin was not observed in the wild type. Thus, as predicted by the reviewer, these results suggest that ubiquitinated Dome cannot be processed to the degradation step in the *drn* mutant because of a lack of Drn activity. However, the large clumps of Dome were not stained by typical endosome or lysosome markers.
Therefore, although the large clumps of Dome were stained by an anti-ubiquitin antibody, they were atypical intracellular compartments related to endosomes. To describe these results and speculations, we have modified and added the following sentences and presented the results in Fig. 5L–M” in our revised manuscript. We have also modified the Abstract to include these new results.

Modified sentences:

Results; Drn is required for endocytic trafficking of the Dome receptor; second paragraph, line 5:
Previously, Dome was found to accumulate in ubiquitinated cargoes when endocytosis was impaired (Tognon et al., 2014). Hence, we assessed whether these vesicles containing Dome-GFP were stained with an anti-ubiquitin antibody that could recognize mono- and polyubiquitin. However, we did not detect colocalization between Dome-DFP and ubiquitin in the wild-type cells (Fig. 5L-L”). In the epidermal cells of drn^2 homozygotes, Dome-GFP was markedly higher than that in wild-type embryos in all cases, as observed in images of Dome-GFP obtained at the same gain of signal amplification (Fig. 5B). In images obtained using reduced gain of signal detection, Dome-GFP was observed as larger clumps located near the plasma membrane (Fig. 5B’, B”). To analyze the nature of these clumps, we costained Dome-GFP with markers of representative intracellular compartments, including Pdi (ER), GM130 (*cis*-Golgi), PNA (*trans*-Golgi), Sec5 (exocytic vesicles), Hrs (early endosomes), Rab5 (early endosomes), Rab7 (late endosomes), Rab11 (recycling endosomes), and LAMP1 (lysosomes), in the epidermal cells of drn^2 homozygotes. None of these markers colocalized with Dome-GFP (Fig. 5C–K”). However, large clumps of Dome-GFP often colocalized with ubiquitin (Fig. 5M-M”). Our quantitative analyses revealed that 3.6% of these Dome-GFP clumps colocalized with ubiquitin and 5.3% of ubiquitin-positive vesicles colocalized with Dome-GFP. We speculated that Dome-GFP localizes with ubiquitinated cargos only temporarily because colocalization was observed only in a subset of these vesicles. Nevertheless, as ubiquitination regulates endosomal trafficking and sorting, these results suggest that Dome-GFP accumulates in atypical endosomal compartments in *drn* mutants. Such a defect in endocytosis may cause JAK/STAT signaling to deteriorate, supporting previous suggestions that endocytosis is essential for Dome activation (Devergne et al., 2007; Moore et al., 2020). Additionally, defective endocytosis may prevent the degradation of Dome-GFP in lysosomes of *drn* mutants.

Abstract, line 9:
The absence of Drn resulted in the specific accumulation of Domeless (Dome), the receptor of JAK/STAT signaling, in intracellular compartments, including ubiquitinated cargos. Dome colocalized with Drn in wild-type *Drosophila*. These results suggest that Drn is required for the endocytic trafficking of Dome, which is a crucial step for the activation of JAK/STAT signaling and the subsequent degradation of Dome.

© 2023. Published by The Company of Biologists under the terms of the Creative Commons Attribution License (https://creativecommons.org/licenses/by/4.0/).

Response 2-2:
As pointed out by the reviewer, our description of the reference Tognon et al. (2014) was not precise, although their results did suggest that Dome is ubiquitylated because it accumulated and colocalized with ubiquitin in ESCRT-0 mutants. Hence, in the revised manuscript, we have modified the previous sentence to mention these results more precisely, as shown below.

Added sentences:

Discussion; Drn is specifically involved in the endocytic trafficking of Dome; first paragraph, line 12:
As such intracellular compartments, including aggregated Dome, were stained with an anti-ubiquitin antibody, it was proposed that *Drosophila* Dome is ubiquitinated and its endosomal sorting is controlled by ubiquitination; however, the ubiquitination of Dome was not confirmed biochemically (Tognon et al., 2014).

Response 2-3:
The reviewer suggested an experiment to map the ubiquitylation site(s) on Dome. However, we determined that this experiment should not be considered a part of the revision because it would require an exorbitant amount of work. In addition, information on the ubiquitylation site(s) may not provide crucial information for our study.

3) The localization studies were performed in epidermal cells overexpressing Domeless-GFP, not cells in which Drn physiologically regulates LR asymmetry. Although I fully understand that based on the accessibility of the tissue, the imaging of cells of interest is difficult, I am questioning the conclusions that can be drawn/biological relevance of the observations. Are the regulators of JAK/STAT pathway and membrane trafficking regulators, potential Dome-specific E3 ligase, expressed similarly in epidermal cells than in gut cells? along the same line, the authors raise the apparent discrepancies between the physiological, in vivo, positive role of endocytic pathway in the regulation of JAK-STAT signaling in Drosophila egg chamber (Devergne et al, 2007) and the negative role of endocytosis obtained in vitro in KC167 cells (Vidal et al, 2010). As a developmental cell biologist, I tend to give more weight to the physiological context in vivo than to the cultured cells.

Response:
As pointed out by the reviewer, it would be ideal if our localization experiments could be conducted in the visceral muscles of the midgut, where LR asymmetric development of the anterior gut is dictated. Although we earnestly tried to conduct such experiments, we were unsuccessful in obtaining clear images of visceral muscle cells because of two reasons. First, the midgut exists deep inside the embryo, making it difficult to obtain clear images of intracellular compartments using confocal laser microscopes. Second, the visceral muscles are very thin, also making it difficult to find proper optical sections.
However, in the previous manuscript, we reported that *drn* is expressed in the embryonic epidermis, where we conducted our localization experiments. A positive requirement of the endocytosis of Dome found in our study has also been reported in a study conducted on the *Drosophila* egg chamber (Devergne et al., 2007). Thus, so far, we have not found any discrepancies among the results obtained in various *Drosophila* tissues. Therefore, in the revised manuscript, we did not experimentally address these issues.

4) The schematical representation depicted in Fig. 6 is inappropriate in my opinion: what are the evidence that Drn localizes in internal vesicles inside LE (multivesicular body?)? The ubiquitin moiety is removed prior to incorporation into MVBs. There is no evidence that Domeless is Ubiquitinated in the absence of Drn? And there no evidence that it is the internalized Domeless-GFP that accumulates in the ‘unknown compartments?

Response:
Thank you very much for the excellent advice to improve our summary diagram. In the revised manuscript, we have reported that Dome accumulated in large clumps was ubiquitinated in the *drn* mutant [please see our response to comment (2) of this reviewer]. Therefore, in the

© 2023. Published by The Company of Biologists under the terms of the Creative Commons Attribution License (https://creativecommons.org/licenses/by/4.0/).

revised manuscript, we have kept the diagrams of ubiquitinated Dome. However, as pointed out by the reviewer, ubiquitinated Dome should not be incorporated in MVBs. Hence, we removed Dome from the intraluminal vesicles in MVBs and placed ubiquitinated Dome at the limiting membrane of MVBs. As mentioned by the reviewer, we have replaced “unknown compartments” with “compartments yet identified” in the revised manuscript. To explain these points, we have modified the legend for Fig. 6 as follows.

Modified sentences:
Legend for Fig. 6, line 7:
Moreover, the failure of endocytosis leads to the abnormal accumulation of Dome in atypical endocytic compartments containing ubiquitin, which are yet to be identified.

Minor comment

1) Figure 1G: difference between AMG defect (middle) and FG + AMG defect (right panel) is difficult to see/apprehend for non-expert, schematical representations would help.

Response:
The schematic representations requested by the reviewer have been added to Fig. 1G. To describe them, we have added the following sentence.

Legend for Fig. 1:
Schematic diagrams representing LR defects are presented below.

2) Figure 3K: the rescue of Stat92E 06346 mutant phenotype by misexpression of drn is interesting. It would be worthwhile to elaborate on how the authors envisage that Drn can rescue Stat92E in molecular terms. The term epistasis is employed. To my knowledge, this term can be used when testing two mutant genes, not for misexpression of a gene. Perhaps to test for epistasis, analyzing the double mutant context, drn together with dome/upd/Stat92E or hop will be informative? One prediction is that for two genes involved in the same pathway, in case of null genetic alleles, the phenotype of the double mutant should be non- additive.

Response:
As pointed out by the reviewer, we also found this observation interesting. In our previous manuscript, we attempted to explain this observation in the sentence referring to “epistasis.” Unfortunately, we realized that this explanation was unclear. To avoid such ambiguity, we have added an elaborate discussion to the revised manuscript, as suggested by the reviewer. To explain our interpretation, we have answered the reviewer’s question. To our knowledge, we can induce overexpression to assess epistasis between two genes. However, one gene needs to be overexpressed in a null condition of the other gene in order to test the latter’s requirement for the former’s function. In our experiment, the *Stat92E* mutant needs to be in a null condition, but it is known that *Stat92E* has a maternal contribution (Hou et al., 1996; Li et al., 2003; Tsurumi et al., 2011). Therefore, embryos homozygous for *Stat92E* have a maternal supply of *Stat92E* that is presumably sufficient to support the activity of *drn* overexpressed in these embryos. We hence speculated that defects associated with *Stat92E* were restored through *drn* overexpression. To further explain our interpretation, we have modified the text, as mentioned below.
In addition, the reviewer has suggested a set of experiments involving double mutants.

However, we believe that our overexpression experiment would be more informative. Therefore, we have not conducted the suggested experiments.

Results; *drn* is a general component of the JAK/STAT signaling pathway; second paragraph, line 5:

Modified sentences:

This result can be explained by a previous finding that *Stat92E* exhibits a maternal effect (Hou et al., 1996; Li et al., 2003; Tsurumi et al., 2011). We speculated that the supply of maternal *Stat92E* to *Stat92E*^{06346} mutant embryos is sufficient to support the activity of overexpressed

© 2023. Published by The Company of Biologists under the terms of the Creative Commons Attribution License (https://creativecommons.org/licenses/by/4.0/).

drn, which can consequently rescue LR defects of these embryos.

Previous sentences:

This result was not due to epistasis between *drn* and *Stat92E*, since embryos homozygous for *Stat92E*^{06346} still have residual *Stat92E* activity, which is maternally supplied (Hou et al., 1996; Li et al., 2003; Tsurumi et al., 2011).

Response to Reviewer 2

Minor comments

1) The authors may speculate why only a portion (~30%) of drn mutants show laterality defects in AMG and/or FG (for example, drn2 in Fig. 1F).

   Response:
   Thank you very much for your valuable comments. In LR asymmetry formation, complete randomization of LR asymmetry results in 50% abnormality. Therefore, the prevalence of defects in LR asymmetry (30%) is not very low. In addition, *drn*^{GS12294} showed LR defects of the anterior gut at a prevalence of 42.5%, which largely corresponds to LR randomization. Therefore, we have not discussed this issue in our revised manuscript.

2) I believe that the image shown in Fig. 5B” is lower gain than its counterpart A’. This should be mentioned clearly in the legend to avoid misunderstanding.

   Response:
   As suggested by the reviewer, we have added a description stating that they are lower gain images to the legend for Fig. 5.

   Added sentences:
   Gain to capture was the same for images in A, A’, and B. Images in B’ and B” use a lower gain to visualize intracellular aggregations and avoid signal saturation. A’ and B” are magnified views of A and B’.

3) Dome protein was accumulated in the epidermis of drn2 mutant. Were Dome accumulates localized in lysosome as speculated by the authors? It would be nice to see co-staining with LAMP1 in Fig. 5.

   Response:
   As suggested by the reviewer, we performed double staining of Dome and LAMP1. We found that Dome rarely colocalized with LAMP1 in the *drn* mutant. Therefore, large clumps of Dome were not localized in lysosomes. On the other hand, to address a comment of reviewer 1 (major comment 2), we examined whether Dome colocalized with ubiquitin, a marker of endosomes, and noted colocalization between them in the *drn* mutant. Hence, we speculated that Dome is incorrectly transported to some atypical intracellular compartments that are related to endosomes but not labeled by Hrs, Rab5, Rab7, Rab11, and LAMP1. We have added these results to Fig. 5. To describe our results, we have added the following sentences to the Results section and the legend of Fig. 5.

   Added sentences:
   Results; Drn is required for endocytic trafficking of the Dome receptor; second paragraph, line 19:
   However, large clumps of Dome-GFP often colocalized with ubiquitin (Fig. 5M-M”). Our quantitative analyses revealed that 3.6% of these Dome-GFP clumps colocalized with ubiquitin and 5.3% of ubiquitin-positive vesicles colocalized with Dome-GFP. We speculated that Dome-GFP localizes with ubiquitinated cargos only temporarily because colocalization was observed only in a subset of these vesicles. Nevertheless, as ubiquitination regulates endosomal trafficking and sorting, these results suggest that Dome-GFP accumulates in atypical endosomal compartments in *drn* mutants.

© 2023. Published by The Company of Biologists under the terms of the Creative Commons Attribution License (https://creativecommons.org/licenses/by/4.0/).

Legend for Fig. 5:
The expression of *UAS-dome-GFP* was ubiquitously driven by *da-GAL4* in wild-type (L-L”) and *drn*^{2} mutant (M-M”) embryos. Dome-GFP and ubiquitinylated proteins were detected by anti-GFP (green, L, L”, M, and M”) and anti-ubiquitin (Ub) (magenta, L’, L”, M’, and M”) antibody staining, respectively, in epidermal cells. L” and M’’ are merged images of L-L’ and M-M’, respectively. White arrowheads denote vesicles showing colocalization between aggregated Dome-GFP and Ub in *drn*^{2}. Scale bar: 5 μm.

4) Drn protein localization was studied in the epidermis in Fig. 4 and Fig. 5. I wonder if Drn is similarly localized in the anterior gut cells.

Response:
Thank you very much for the excellent comment. Unfortunately, it was too difficult to obtain clear images of the midgut visceral muscles for two technical reasons. Since the midgut exists deep within the embryo and visceral muscles are very thin, it is difficult to find proper optical sections and obtain clear images using confocal laser microscopes. Given these technical difficulties, we were unable to address this point.

Response to Reviewer 3

Minor comments

1) for clarity, please mark Left and Right on pictures and graphs

Response:
Thank you very much for helping us improve the clarity of our manuscript. We added “Left” and “Right” to the pictures in the revised manuscript. To explain these modifications, we have added the following sentence to the legends for Fig.1 and Fig. 2.

Legend for Fig. 1, last sentence:
L and R represent the left and right sides of embryos, respectively.

Legend for Fig. 2, last sentence:
In E and F, L and R represent the left and right sides of embryos, respectively.

2) results p3 line21 : ‘located on the right side of the midline’, do the authors mean ‘left side’ (fig1A) ?

Response:
We agreed that our explanation was insufficient. As embryos were observed from the ventral side, our left side would be the right side of the embryo, as described in our previous version. For clarity, we have added “Left” and “Right” to all figures, as mentioned in our response to comment (1) of this reviewer.

3) the drn2 excision allele is proposed to be a null mutation, however, it has a weaker phenotype than the original P insertion allele (fig1F)

Response:
We agree with this comment. As pointed out by the reviewer, the phenotype of *drn*^{2} was not particularly strong among *drn* mutants. Therefore, we have described *drn*^{2} as a loss-of-function allele instead of a null allele in the revised manuscript. The modified sentences are as follows:

Results; *drn* mutations affect LR-asymmetric gut morphogenesis in *Drosophila*; second paragraph, line 13:
To genetically characterize *drn* further, we generated deletion mutant alleles of *drn* through imprecise P element excision. In *drn*^{1}, the deduced initiation codon and 5′ portion of the

© 2023. Published by The Company of Biologists under the terms of the Creative Commons Attribution License (https://creativecommons.org/licenses/by/4.0/).

coding region were deleted from the *drn* alternative RNA products CG45050-RB, -RC, -RD, -RE, -RF, and -RG (Fig. 1D). In *drn*^{2}, the deduced initiation codon and most coding sequences were deleted from all *drn* alternative RNA products, suggesting that *drn*^{2} is a loss- of-function mutant of *drn* (Fig. 1D).

4) it is not completely clear what the authors call a ‘defect in AMG’ (p. 4). Could you please clarify?

Response:
When the orientation of FG is correct but the joint between FG and PV is on the opposite side, an LR reversion occurs at the start point of the AMG, which is characterized as an LR defect in the AMG. To clarify this point, we have modified a sentence, as mentioned below.

Modified sentence:

Results; *drn* mutations affect LR-asymmetric gut morphogenesis in *Drosophila*; second paragraph, line 20:
These mutant embryos had LR defects in both the FG and AMG or in the AMG alone (orientation of the FG was correct, but the joint between the FG and PV was on the opposite side); however, they never had defects in the FG alone (Fig. 1F, G).

Previous sentence:
These mutant embryos had LR defects in both the FG and AMG or in the AMG alone, but never in the FG alone (Fig. 1F, G).

5) it would be a nice confirmation if authors could recapitulate the phenotype driving drn-RNAi in CVMU cells

Response:
We agree with this proposal. However, we found that UAS-RNAi did not work well in *Drosophila* embryos, which has been reported in various cases. Therefore, we did not conduct this experiment.

6) p5 , first paragraph: have the authors tried to invalidate Jak/Stat activity in CVMU cells using RNAi ?

Response:
Because of the technical reason described in comment (5) of this reviewer, we did not conduct this experiment.

7) fig3F,F’ : embryos do not seem to be properly oriented along the DV axis

Response:
Thank you very much for your excellent advice to improve the clarity of our manuscript. As suggested by the reviewer, we have adjusted the orientation of these embryos.

8) p6, drn-antibodies : did the authors tested antibody reactivity upon drn overexpression?

Response:
We found that the overexpression of *drn* would not be very informative to determine whether our antibody specifically detects endogenous Drn protein. As our RNAi experiment against *drn* assured the specificity of our antibody to detect endogenous Drn, we did not conduct this experiment.

9) trafficking (figs4&5): this part is missing quantification to make a strong point regarding Drn localization in endocytic compartments. Also, correlation coefficients (e.g., Pearson’s and Manders) should be used to confirm co-localization.

© 2023. Published by The Company of Biologists under the terms of the Creative Commons Attribution License (https://creativecommons.org/licenses/by/4.0/).

Response:
We agree with this comment. To address this point, we tried to quantify the percentages of colocalization between Drn and endocytic compartments. However, we found that such quantitation is very difficult because of the few potential colocalization examples. This made it difficult to determine an appropriate threshold of Drn and marker signals for judging colocalization. Therefore, we did not include such quantitation in order to avoid erroneous evaluation during data processing.

On the other hand, Dome showed a relatively high ratio of colocalization with Drn and ubiquitin, allowing us to define signal threshold levels and quantify their percentage. To describe the quantitation procedure, we have added a new subheading to the Materials and Methods section. However, the distribution patterns of Dome- and Drn-positive vesicles were very different. Therefore, we considered that correlation coefficients would not be appropriate for our analysis, and we did not include them.

Added sentences:
Materials and Methods; Quantitative analyses of colocalizations in intracellular vesicles: The same threshold was set for each pair of images (two channels representing respective markers) using ImageJ. Using the Analyze Particles function, particles larger than five-pixel units were defied as the region of interest (Roi). Roi sets from two channels of an image were compared. Each Roi overlapping between two channels was defined as a particle demonstrating colocalization and was used for calculating the colocalization rate between two markers. The number of Rois showing colocalization against the total number of Rois was calculated as the percentage of colocalization.

10) Introduction line 11: Speder et al 2006 should be acknowledged when referring to the identification of myo31DF/myo1D. As for nomenclature, myo1D should be preferred as it is now the standard name for the gene in both flies and vertebrates.

Response:
We have added the study of Speder et al. (2006) to the references in our revised manuscript. The gene name has been changed to *MyoID*, as suggested by the reviewer.

11) Introduction p3 last line : Devergne et al 2002 should be acknowledged when referring to Dome identification

Response:
Thank you very much for pointing out this omission. The reference has been added as Ghiglione et al. (2002).

12) Introduction line 18 : Ligoxygakis et al, should not be cited as its conclusions (maternal origin of gut LR asymmetry) have been clearly and elegantly refuted by Hayashi et al. 2005.

Response:
Thank you very much for your valuable suggestion. We have replaced this with the study of Hayashi et al. (2005).

Second decision letter

MS ID#: DEVELOP/2022/201224

MS TITLE: The Drosophila AWP1 ortholog Doctor No regulates JAK/STAT signaling for left-right asymmetry in the gut by promoting receptor endocytosis

AUTHORS: Yi-Ting Lai, Takeshi Sasamura, Junpei Kuroda, Reo Maeda, Mitsutoshi Nakamura, Ryo Hatori, Tomoki Ishibashi, Kiichiro Taniguchi, Masashi Ooike, Tomohiro Taguchi, Naotaka Nakazawa, Shunya Hozumi, Takashi Okumura, Toshiro Aigaki, Mikiko Inaki, and Kenji Matsuno

© 2023. Published by The Company of Biologists under the terms of the Creative Commons Attribution License (https://creativecommons.org/licenses/by/4.0/).

ARTICLE TYPE: Research Article

I am happy to tell you that your manuscript has been accepted for publication in Development, pending our standard ethics checks.

Reviewer 1

Advance summary and potential significance to field

In this article, the authors report the identification of the ubiquitin-binding protein Doctor no (Drn)/AWP1 as a novel regulator of left-rigth asymmetric gut morphogenesis. Understanding in molecular and cellular terms how LR asymmetry established is of fundamental importance in developmental biology.

Comments for the author

As mentioned in my previous review, the first part of the manuscript describing the identification and characterization of the function of the ubiquitin-binding protein Doctor No (Drn)/AWP1 as a regulator of left asymmetric gut morphogenesis is compelling. The effect of loss of Doctor No (Drn) on Dome distribution is also convincing.
In this revised version, the authors have repeatedly attempted to answer my concerns about the subcellular localization of Dome. They provide arguments as to why they did not perform internalization assays (lack of available reagents) that would have tested the involvement of Drn in receptor endocytosis, and why they did not mutate the cytoplasmic domain of Dome aiming to prevent its post-translational modification by Drn, nor to image in the tissue where Drn is reguate L/R asymmetry (due to the tickness of the tissue and access to imaging).
While I agree that mutation of Dome may be beyond the scope of the paper, I am still disappointed that the authors did not address the first issue that would have supported their main claim (promote receptor endocytosis). However, and as requested, the authors did perform quantitative analyses to determine the extent of Dome colocalization with endocytic markers and ubiquitinated cargoes. They consistently report very low amounts of colocalization (up to 5-6%). They interpret the low percentage of colocalization as a reflection of short time residence in the given compartments, an hypothesis that could be addressed by time lapse microcopy. Of note, the images are of low magnification, so the signals could be closely juxtaposed without actually colocalizing. The low colocalization is close to background (the same analysis on images with one of the channels rotated by 90° may give a similar colocalization percentage) and is therefore, in my opinion, not a strong argument supporting the author's claims.
I am therefore afraid that on the cell biology side, the study is substandard and remains insufficiently well documented to be accepted for publication.

Reviewer 2

Advance summary and potential significance to field

Drosophila exhibits laterality in the anterior and posterior regions of the gut. However, the both regions are regulated by different genetic pathways. While the genetic and cellular mechanism underlying the laterality of posterior region of the gut has been studied in detail, the anterior region of the gut remain unexplored. In this paper, the authors have performed a genetic screen and have searched for mutants defective in the laterality of the anterior gut. They have identified two mutants, both of which are mapped to a single gene drn. Genetic data suggest that Drn is a component of JAK/STAT signaling required for endocytic trafficking of the ligand Dome. In support of this, Dome was accumulated in the epidermis of drn mutant, which show additional phenotypes such as aberrant expression of even-skipped and abnormal trachea branching, typical of JACK/STAT signaling defects. I believe that the paper provides two lines of new information for our understanding of laterality in Drosophila and of JACK/STAT signaling pathway, and these findings deserve publication in Development.

© 2023. Published by The Company of Biologists under the terms of the Creative Commons Attribution License (https://creativecommons.org/licenses/by/4.0/).

Comments for the author

I only had minor comments and in the first place. As far as this reviewer is concerned, the revised paper should be accepted.

Reviewer 3

Advance summary and potential significance to field

The authors have addressed most of the reviewer's comments and the manuscript is clearer.

Comments for the author

The authors have addressed most of the reviewer's comments and the manuscript is clearer.

© 2023. Published by The Company of Biologists under the terms of the Creative Commons Attribution License (https://creativecommons.org/licenses/by/4.0/).
